# A DNA turbine powered by a transmembrane potential across a nanopore

**DOI:** 10.1038/s41565-023-01527-8

**Published:** 2023-10-26

**Authors:** Xin Shi, Anna-Katharina Pumm, Christopher Maffeo, Fabian Kohler, Elija Feigl, Wenxuan Zhao, Daniel Verschueren, Ramin Golestanian, Aleksei Aksimentiev, Hendrik Dietz, Cees Dekker

**Affiliations:** 1 Department of Bionanoscience, Kavli Institute of Nanoscience Delft, Delft University of Technology, Delft, The Netherlands; 2 Department of Bioscience, School of Natural Sciences, Technical University of Munich, Garching, Germany; 3 Munich Institute of Biomedical Engineering, Technical University of Munich, Garching, Germany; 4 Department of Physics, University of Illinois at Urbana-Champaign, Urbana, IL USA; 5 Max Planck Institute for Dynamics and Self-Organization, Göttingen, Germany; 6 Rudolf Peierls Centre for Theoretical Physics, University of Oxford, Oxford, UK; 7 Present Address: Department of Chemistry, KU Leuven, Leuven, Belgium; 8 Present Address: The SW7 Group, London, UK

**Keywords:** Nanopores, DNA nanomachines

## Abstract

Rotary motors play key roles in energy transduction, from macroscale windmills to nanoscale turbines such as ATP synthase in cells. Despite our abilities to construct engines at many scales, developing functional synthetic turbines at the nanoscale has remained challenging. Here, we experimentally demonstrate rationally designed nanoscale DNA origami turbines with three chiral blades. These DNA nanoturbines are 24–27 nm in height and diameter and can utilize transmembrane electrochemical potentials across nanopores to drive DNA bundles into sustained unidirectional rotations of up to 10 revolutions s^−1^. The rotation direction is set by the designed chirality of the turbine. All-atom molecular dynamics simulations show how hydrodynamic flows drive this turbine. At high salt concentrations, the rotation direction of turbines with the same chirality is reversed, which is explained by a change in the anisotropy of the electrophoretic mobility. Our artificial turbines operate autonomously in physiological conditions, converting energy from naturally abundant electrochemical potentials into mechanical work. The results open new possibilities for engineering active robotics at the nanoscale.

## Abstract (web summary)

A nanoscale DNA origami turbine is shown to perform mechanical rotation by directly harvesting transmembrane potential energy from an ion-concentration gradient across a solid-state nanopore. The direction of rotation is set by the designed chiral twist in the turbine’s blades.

## Main

At the heart of any active mechanical system is an engine, which converts one type of energy, typically chemical or electrical, into mechanical work. In biological systems, such work is done by motor proteins such as kinesin^1^, the bacterial flagella motor^2^ and F_o_F_1_-ATP synthase^3,4^. In the latter, electrochemical potential energy from a concentration gradient of ions is converted into mechanical rotary motion of the F_o_ motor, which drives the F_1_ rotary complex to catalyse the synthesis of ATP, the molecule that provides free energy for many cellular processes. Despite the extensive knowledge and success of building rotary engines of sizes spanning many orders of magnitude on the macroscale, designing, building and demonstrating functioning artificial nanoscale counterparts of these sophisticated biological motors has proven challenging.

The critical step of building such nanoscale rotary engines is to demonstrate their ability to transduce local free energy continuously and autonomously into designed mechanical motion and useful work. Previous work led to multiple designs of rotary assemblies^5–8^, and established a certain level of directed motion as an external operator manually cycled environmental conditions^9^ such as light and temperature^10,11^, chemical compounds^12,13^ or alternate macroscale electric fields^14^. Molecular dynamics (MD) simulations have shown the conceptual feasibility of using a DNA helix to convert electric field into torque^15^; however, experimental demonstration of a rotary mechanism programmed for sustained conversion of a transmembrane electric potential into the mechanical rotation had not been achieved.

Here, we demonstrate a bottom-up designed DNA nanoturbine that is powered by a nanoscale hydrodynamic flow inside a nanopore. It contains a central axle decorated with three blades arranged in a chiral configuration, either left or right handed. The turbine has a height of 24 or 27 nm, comparable to the 20-nm-tall ATP synthase. The turbine’s stator is provided by a solid-state nanopore in a 20-nm-thickness silicon nitride membrane. Using single-molecule fluorescence, we monitor the rotation of the nanoturbine driven by either a direct current (DC) voltage or a transmembrane ion gradient, which mimics the working environment of rotary motors in biological cells. As we demonstrate below, the nanoturbine can drive a long DNA bundle as a hydrodynamic load into sustained rotary motion of up to 10 revolutions s^−1^, equivalent to delivering tens of piconewton nanometres of torque, which compares well with the ~50 pN nm torque that can be generated by natural ATP synthase^16,17^.

## Design of DNA nanoturbines

**Fig. 1:**
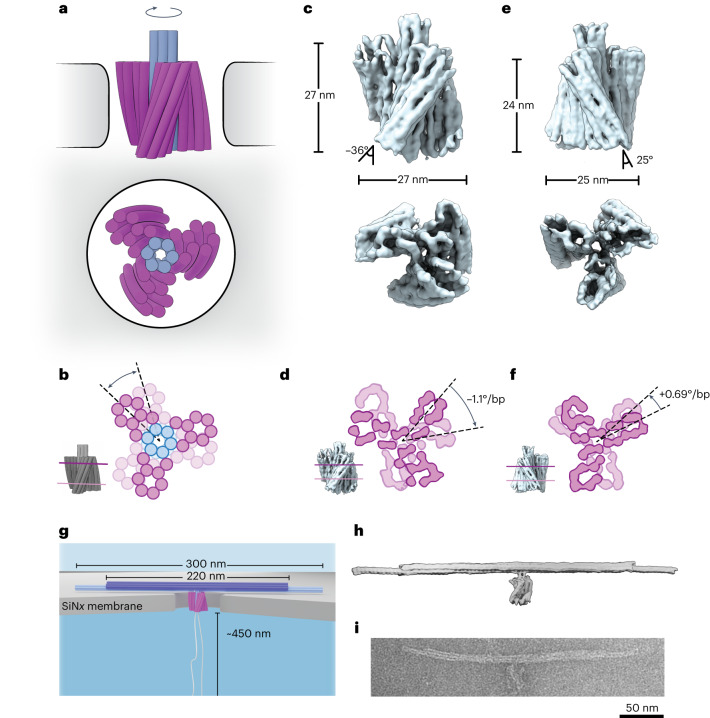
Design of a nanopore-powered DNA origami turbine. **a**, Schematic of a right-handed DNA turbine docked into a nanopore (side view at top; axial view at bottom). **b**, Two cross-sections of the DNA turbine highlighting the designed twist. **c**, 3D electron density map of the right-handed DNA turbine determined via single-particle cryo-EM (side and bottom views; see also Supplementary Fig. 2). **d**, Cross-sections at the top and the bottom of the 3D cryo-EM reconstruction right-handed turbine, highlighting the right-handed twist density of −1.1° bp^−1^. **e**,**f**, The same as **c**,**d** but for the left-handed DNA turbine, highlighting the twist density of +0.69° bp^−1^. **g**, Schematic of a right-handed DNA turbine with its load, a 300-nm-long DNA bundle with the middle 220 nm reinforced with 16 DNA helices instead of 6 helices, and a 900 nm looped leash docked onto a solid-state silicon nitride nanopore. **h**, SNUPI-simulated structure of the right-handed DNA turbine with a DNA bundle attached as a load (leash excluded). **i**, Negatively stained transmission electron micrograph of a typical right-handed DNA turbine with the load.

Our DNA nanoturbine is a multilayer DNA origami structure containing an intentionally designed chiral twist (Fig. 1a,b). The structure consists of 30 double-stranded DNA helices, each 72 base pairs (bp) in length on average, where the six parallel central helices form an axle, and the three eight-helix blades are obliquely attached to the axle and symmetrically spaced at 120° angles across the circumference. The chiral twist in the blades of the turbine is induced by adjusting the number of base pairs between each staple crossover away from the one-per-7 bp value required for an achiral structure^18^, yielding a strongly right-handed twisted structure for an 8 bp crossover density and a left-handed twisted turbine for 6.5 bp spacings on average, while maintaining a good folding yield of the structures (Supplementary Figs. 1–5). The objects were self-assembled as described previously^19^ (for details, see Methods). We used single-particle cryogenic electron microscopy (cryo-EM) to determine three-dimensional (3D) electron density maps of the right-handed and left-handed turbine structures (Fig. 1c,e and Supplementary Figs. 6–9). The cryo-EM reconstructions showed the desired structural features such as the three blades and the axle, and revealed the twisted orientation of the blades. The twist and the blade angles were measured from the cryo-EM data, yielding a −1.1° bp^−1^ twist density and a blade angle with respect to the turbine axis of −36° for the right-handed structure, and +0.69° bp^−1^ and +24° for the left-handed structure (Fig. 1d,f; for details see Methods and Supplementary Fig. 10). The turbine structures were 27 nm and 24 nm tall (right and left handed, respectively) and had diameters of 27 nm and 25 nm, respectively.

## Unidirectional rotation of DNA turbines driven by a salinity gradient or transmembrane voltage

To demonstrate that the turbines can generate torque and work, we docked the structure into a nanopore, and optically monitored rotations at the single-molecule level. To create a hydrodynamic load as well as to hold the turbine in the nanopore and to facilitate optical tracking using super-resolution microscopy, we attached a 300-nm-long DNA six-helix bundle as a crossbar featuring a reinforced 220-nm-long central 16-helix bundle segment (Supplementary Fig. 12) to the top part of the turbine axle as one continuous rigid body (Fig. 1g). One end of the DNA bundle was labelled with ten Cy3 fluorophores to allow continuous monitoring of its motion by fluorescence microscopy at 5 ms temporal resolution and sub-diffraction-limit localization precision (Methods). A 900-nm-long loop of nicked double-stranded DNA was engineered to extend from the bottom of the axle and act as a leash guiding the insertion of the turbine into the nanopore during the docking process (Fig. 1g). Coarse-grained simulations (SNUPI^20^) were used to analyse the structural rigidity of the integrated design (Fig. 1h). Proper folding of the entire assembly was verified by negative-stained transmission electron microscopy (Fig. 1i; for details see Methods). An array of 50-nm-diameter nanopores was fabricated in 20-nm-thickness silicon nitride membranes using electron-beam lithography and reactive ion etching (Methods) and characterized with transmission electron microscopy (Supplementary Fig. 13).

**Fig. 2:**
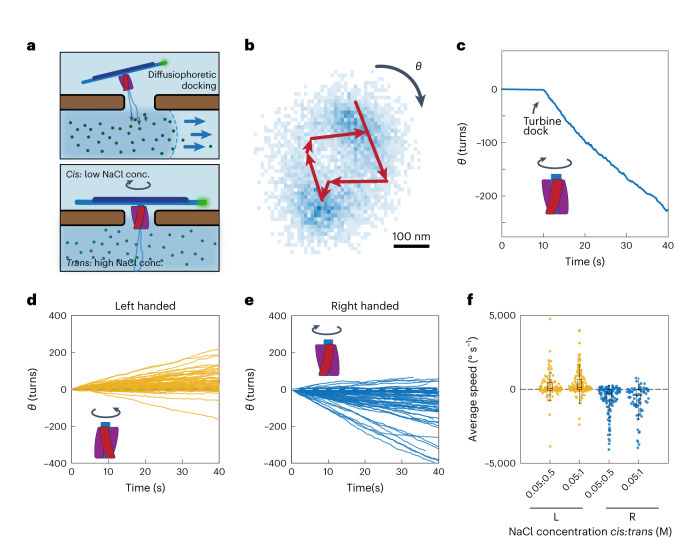
Sustained unidirectional rotation of DNA origami turbines in a salt gradient. **a**, Schematic of a DNA turbine docking onto a nanopore by diffusiophoresis. **b**, Typical heatmap (blue pixels) of obtained centres of fluorophores at the tip of DNA bundle from single-particle localizations from 8,000 frames (Methods) and example trajectory of six subsequent positions of the labelled tip (red), which shows clear directional rotation. **c**, Typical cumulative angle versus time for a right-handed turbine in 50 mM:1 M NaCl, showing a sustained rotation of hundreds of turns. **d**,**e**, Cumulative angle versus time of left-handed (**d**) and right-handed (**e**) turbines for a NaCl concentration gradient of 50 mM:1 M (*n* = 77 and 151, respectively). **f**, Average rotation speed of left- and right-handed turbines in transmembrane NaCl concentration gradients of 50 mM:500 mM and 50 mM:1 M (*n* = 98, 141, 124 and 74, respectively). In all box plots: centre line, median; box limits, upper and lower quartiles; whiskers, 1.5 × interquartile range. Source data

Our single-molecule observations show that the DNA turbine can drive a unidirectional rotation of the load under a transmembrane gradient of ion concentration. Initially, both compartments of the flow cell were filled with 50 mM NaCl buffer, and the DNA turbines were added to the *cis* compartment. Subsequently, a higher concentration of NaCl (0.5–3 M) was flushed into the *trans* compartment (Fig. 2a), causing the DNA turbines to move towards the nanopores by diffusiophoresis, which led to the insertion of the turbines into the nanopores. As the leash guided the initial insertion of the large structure^21^, the turbine oriented itself upon docking in the designed orientation, where the extended crossbar bundle prevented the turbine from translocating through the nanopore. After docking, we tracked the rotary motion of the DNA bundle by monitoring the position of the fluorophores, which were located at one end of the bundle (Supplementary Fig. 14).

As shown in Fig. 2, we observed clear, directed rotation of the turbine particles. Figure 2b shows the rotary motion of the bundle end, which can be traced to follow a circular path over time. Figure 2c shows the cumulative angular displacement corresponding to the example in Fig. 2b, which continues over 200 clockwise rotations over the full period of observation (40 s in this case). Figure 2d,e shows typical data for 228 turbines, where right- and left-handed turbines led to, respectively, upwards and downwards linear angular rotation curves. The linearity of these curves (and the corresponding superlinear mean-square angular rotation curves; Supplementary Fig. 15) is direct evidence of a driven motion. The data show that the DNA turbines can exert a substantial torque on the DNA load bundle and drive it into sustained unidirectional rotary motion.

Importantly, we find that the designed chirality sets the rotation direction. The turbine variant with left-handed turbine blades displayed preferentially anticlockwise rotations (as viewed from the *cis* side). In contrast, the turbines with right-handed turbine blades showed almost exclusively clockwise rotations. These data indicate that the designed chirality controls the rotation direction. We determined the average angular velocity in different salt-gradient conditions (Fig. 2f and Supplementary Fig. 16). The velocity directions corresponded well to the designed chiralities of the structures, and the angular velocities were distributed with noticeable spread, with maximum values as high as ~10 revolutions s^−1^. We attribute the spread in the velocity distribution to heterogeneity in the local interactions between the DNA structure and the silicon nitride surface and to potential deformations in the DNA turbine crossbar that can modulate the rotation speed^22^.

**Fig. 3:**
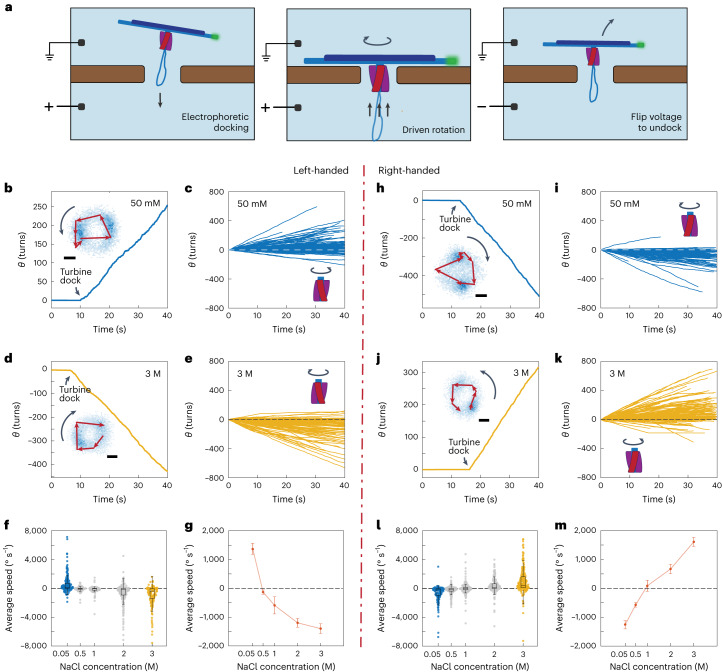
Sustained unidirectional rotation of DNA origami turbines in an applied field. **a**, Schematic of a DNA turbine docking into and undocking from a nanopore on applying a transmembrane voltage. **b**, Typical cumulative angle versus time for a left-handed turbine for a 100 mV bias voltage in 50 mM NaCl, showing a sustained rotation over hundreds of turns. Inset: corresponding heatmap (blue pixels) of single-particle localizations for the tip of the DNA bundle with an example trajectory of the labelled tip overlaid. **c**, Cumulative angular-displacement curves for left-handed turbine-driven DNA bundles as in **b** but for *n* = 210 turbines. **d**,**e**, The same as **b**,**c** but in 3 M NaCl electrolyte (*n* = 159). **f**, Average rotation speed for left-handed turbines in NaCl concentrations of 50 mM, 500 mM, 1 M, 2 M and 3 M (*n* = 198, 77, 86, 252 and 150 respectively). **g**, Mean rotary speed of left-handed turbines for various buffer salt concentrations (Supplementary Fig. 8). Error bars are s.e.m. **h**–**m**, The same as **b**–**g** but for right-handed DNA turbines (*n*_*i*_ = 174, *n*_*k*_ = 298, *n*_*l*_ = 116, 252, 164, 200 and 260. respectively). In all box plots: centre line, median; box limits, upper and lower quartiles; whiskers, 1.5 × interquartile range. All scale bars are 100 nm. Source data

Subsequently, we operated the DNA turbines under a transmembrane voltage that was applied across the compartments at an equal 50 mM NaCl concentration. Immediately after applying the transmembrane voltage (100 mV, Fig. 3a,b), docking and rotary motion of DNA turbines was observed—see Fig. 3b,h for typical traces of a left-handed and a right-handed DNA turbine, respectively. Clear circular trajectories were obtained, indicating a sustained and constant rotary motion over time, very similar to the trajectories observed in the ion-gradient-driven experiments. The extracted rotational velocities of the DNA load bundle showed driven rotary motion, with again predominantly the same rotation direction depending on the chirality of the turbine variant under study, as can be seen in Fig. 3c,i. From the rotational speed of the DNA beam, we estimated the torque of our DNA turbine (Supplementary Section 1 and Supplementary Fig. 20) as tens of piconewton nanometres. As a control, we also tested an approximately non-chiral, straight version of the turbine with the same crossbar load (Supplementary Fig. 18), which was assembled by removing the residual twist in the blades^18^. For this variant, no preferred rotational directionality was observed, while some residual rotation without preferred directionality was observed due to the self-organization of the DNA crossbar^21^ (Supplementary Fig. 18).

## Direction reversal under a transmembrane voltage

To our surprise, we found that we can control the rotational direction of the DNA turbines by the ionic strength of the buffer. When the voltage-driven experiments were performed in a high-salt buffer containing 3 M NaCl instead of dilute 50 mM, the directionality of the rotary motion reversed for the same turbine. This phenomenon occurred for both turbine chiralities, as shown in Fig. 3d,e,j,k (and corresponding mean square displacement, MSD, plots in Supplementary Fig. 19). By titrating the salt concentration from 50 mM to 3 M, we observed that the average rotation speed changed from positive to negative values for the left-handed turbines, that is, rotations changed from anticlockwise to clockwise (and a reverse crossover occurred for right-handed turbines), with a crossover at ~0.5–1 M for both chiralities—see Fig. 3g,m (and corresponding histograms for the speed distribution in Supplementary Figs. 21 and 22). These observations point to the appearance of strong ionic effects on the water flow when the turbine operates in a high-ionic-strength environment.

To understand this rotational reversal induced by different ionic strengths, we first sought insights from continuum theory. As a model for the turbine blade, we consider a rigid cylindrical DNA rod that is held in a fixed vertical position in a wide nanopore, oriented at an angle of *θ* with respect to the *z* axis (cf. Supplementary Section 1). This rod has a hydrodynamic mobility **M**_h_ that is anisotropic, as a rod moves twice as fast through a liquid along its length as perpendicular to it^23^. Similarly, the electrophoretic mobility **M**_el_ of the rod, which describes its freely suspended motion in an applied electric field **E**, is anisotropic^23^. Notably, **M**_el_ is controlled by the electrical double layer, which changes with the ionic strength of the solution^24,25^. The combination of **M**_h_ and **M**_el_ determines the in-plane force on a turbine blade in a nanopore, which will drive the rotation. We can derive an expression for the in-plane velocity component *v*_*x*_ (Supplementary Section 2) as follows:

$${v}_{x}=\frac{\frac{1}{2}{M}_{\mathrm{el},\parallel }{E}_{z}\sin 2\theta }{{\rm{co}}{{\rm{s}}}^{2}\theta +\left(\frac{{M}_{{\mathrm{h}},\perp }}{{M}_{{\mathrm{h}},\parallel }}\right){\rm{si}}{{\rm{n}}}^{2}\theta }\Bigg(\frac{{M}_{{\mathrm{h}},\perp }}{{M}_{{\mathrm{h}},\parallel }}-\frac{{M}_{{\mathrm{el}},\perp }}{{M}_{{\mathrm{el}},\parallel }}\Bigg).$$

This equation indicates that the sign of *v*_*x*_, that is, the rotation direction, is determined by the difference between the hydrodynamic and electrophoretic anisotropy ratios. While the hydrodynamic anisotropy ratio *M*_h,⟂_/*M*_h,∥_ is constant at a value of 0.5, the electrophoretic anisotropy ratio *M*_el,⟂_/*M*_el,∥_ can adopt values between 0 and 1, depending on ion concentration and DNA surface charge^25^; for example, *M*_el,⟂_/*M*_el,∥_ decreases from 1 to 0.5 with decreasing ion concentration for moderate surface charges, and adopts even smaller values for high surface charges^25^. Continuum theory thus indicates that a sign reversal of the rotations may occur due to a change in the electrophoretic anisotropy ratio with salt concentration.

**Fig. 4:**
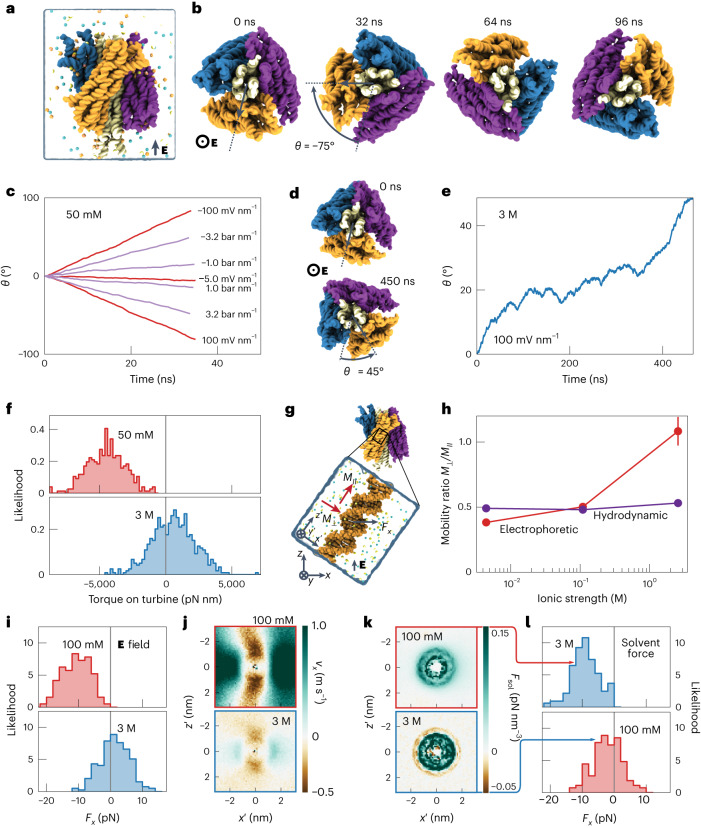
All-atom MD simulation of a DNA turbine rotation. **a**, 4,322,088-atom model of a DNA origami turbine that is depicted using a molecular surface representation (white shaft, multicoloured blades), solvent shown as a semi-transparent surface, and ions (50 mM NaCl, 10 mM Mg^2+^) shown explicitly. **b**, Rotation of the turbine driven by electric field (50 mM NaCl). A 100 mV nm^−1^ field was applied out of the page while restraints prevented the drift and tilting of the turbine. **c**, Rotation angle of the turbine due to an applied field or pressure gradient. **d**,**e**, The same as **b**,**c**, but for simulations at 3 M NaCl. A reversal of the rotation direction is observed. **f**, Torque on the turbine measured by restraining its spin angle. **g**, Single DNA helix as a minimal model for a turbine blade. A field or pressure gradient is applied along the +*z* direction. **h**, Ratio of the electrophoretic and hydrodynamic mobilities for motion perpendicular and parallel to the DNA helical axis observed in simulations of a single DNA helix. Ionic strength is calculated from the average molality observed at distances beyond 6 nm from the helical axis. **i**, Force on DNA orthogonal to the electric field measured from the single-helix simulations. **j**, In-plane solvent flow along the *x* axis in the simulations under an applied electric field. Heat maps depict the average along the helical axis of the DNA. The coordinate system is defined in **g**. **k**, Solvent forces extracted from MD simulation under an applied electric field. **l**, Force on DNA orthogonal to the applied force axis when, as a control, the solvent forces extracted from the low- and high-salt systems are applied to high- and low-salt systems, respectively. The forces were seen to reverse when compared with those in **i**, proving the causal role of the ion distribution. Source data

To elucidate the microscopic mechanism of the torque generation, we performed all-atom MD simulations of the DNA origami turbine submerged in a low-salt electrolyte solution (Fig. 4a). The application of a 100 mV nm^−1^ axial electric field was observed to rotate the turbine by ~120° in 56 ns in the expected direction (left handed about the applied field axis, Fig. 4b and Supplementary Fig. 24, see Methods for details). A water flow induced by a pressure gradient was observed to rotate the turbine in the expected direction, whereas reversing the direction of the electric field or the pressure gradient reversed the rotation direction. The turbine’s rotation speed was found to be determined by the velocity of water molecules moving past the blades of the turbine (Supplementary Fig. 25b)—all indicating a rotation mechanism similar to that of a macroscopic turbine. However, in equivalent simulations of the same DNA turbine carried out in 3 M NaCl, the direction of rotation reversed, while the overall rotation speed decreased (Fig. 4d,e). Furthermore, the average effective torque produced by the turbine under high salt was seen to change sign when compared with the torque under low salt (Fig. 4f). These MD simulation results thus present a striking qualitative resemblance to the experimental results.

To understand the ion-concentration-dependent reversal of the effective torque, we simulated a DNA duplex that was oriented parallel or perpendicular (Supplementary Fig. 26a) to an applied electric field. The observed *M*_el,⟂_/*M*_el,∥_ did indeed change with the measured salt concentration, from about 1 at 2.6 M NaCl to 0.38 at 4 mM NaCl (Fig. 4h and Supplementary Fig. 26b)—a very notable change that would reverse the direction of rotation according to the continuum model. To determine the microscopic mechanism of the rotation reversal, we simulated a DNA duplex that was tilted by 35° relative to the vertically applied field (Fig. 4g), which approximates the inclination of the blades in our turbines. While the duplex’s centre of mass and the spinning angle were harmonically restrained, we measured the effective in-plane force *F*_*x*_ acting on the duplex (Fig. 4i). At low salt a negative force was measured, as expected, whereas at high salt the average in-plane force was reversed to a positive value. As the flow profile around the DNA (Fig. 4j) is driven by the distribution of charges in the solvent, we calculated a 3D map of the solvent forces—the position-dependent average force on solvent voxels applied by the electric field (Methods). The map revealed a slight overcharging of the DNA in the 3 M condition (Fig. 4k), which was nevertheless sufficient to substantially alter the flow of solvent near the DNA (Fig. 4j). To prove the causal role of the ion distribution, we applied the high-/low-salt solvent force on the low-/high-salt systems which, gratifyingly, swapped the effective force distributions (Fig. 4l).

## Conclusions

In summary, we have demonstrated a new type of autonomous active nanomachine, a DNA nanoturbine. We have shown its functionality from the observation of a sustained rotation of a DNA load bundle when the turbines were driven by an electrophoretic or hydrodynamic flow of solvent through a nanoscale pore. Building from our previous work^22,26^, the direction of rotation is now controlled by the designed chirality of the turbine blades as well as by the ionic strength of the buffer. The latter revealed, strikingly, that ionic interactions can even reverse the rotation direction, as the electrophoretic mobility anisotropy changes with buffer salt concentration. Up to tens of piconewton nanometres of torque can be generated by these merely ~25-nm-tall turbines (an order of magnitude smaller than previously reported DNA origami rotors)^22^, numbers that are comparable to those of the F_o_ motor of ATP synthase^17^. The DNA turbines could operate autonomously on physiologically and biologically compatible energy sources (electrochemical potentials) and work at a scale comparable to nature’s own motor proteins, without requiring manual cyclic intervention.

Our work demonstrates a practical approach to designing nanoscale active engines, that is, by using chiral nanoscale structures to leverage transmembrane potential differences through nanoscale hydrodynamic interactions^15^. We believe this to be a powerful new approach to building active nanoscale systems, in particular because of the extensible design with DNA nanotechnology, its flexibility and potential for integration with various biocompatible membrane systems, and its potential compatibility with physiological environments. As in ATP synthase, microscopic reversibility may be exploited in future variants of such turbines to couple this mechanical rotation to uphill chemical synthesis. Nanoscale hydrodynamic turbines constitute biocompatible engines that may serve as a step towards building self-powered nanorobotic systems in environments relevant to molecular biology. Current limitations of our approach include the non-specific interaction between the turbine and the solid surface, the wide variance of the rotational speed and the finite power efficiency. Further work can be done to demonstrate nanorobotic systems based on the turbine concept, specifically the integration of nanoscale motors into biocompatible membranes, improving their efficiency in utilizing ion gradients and integrating them with other passive functional nanomachines to perform more complex tasks.

## Methods

### Nanopore array fabrication

Nanopore arrays are fabricated as reported before^27^. In brief, a 100-nm-thick layer of poly(methyl methacrylate) electron-sensitive resist (molecular weight 950,000, 3% dissolved in anisole, MicroChem Corp) was spin-coated on 20 nm free-standing silicon nitride membranes supported by silicon. Subsequently, the resist was exposed and patterned by an electron-beam pattern generator (EBPG5200, Raith) with 100 keV electron beams. The pattern is developed in a mixture of methyl-isobutyl-ketone and isopropanol with a ratio of 1:3 for 1 min, then stopped in isopropanol for 30 s. The exposed substrates were then etched using reactive-ion etching with fluoroform and argon (200 s, 50 W, 50 sccm of CHF_3_, 25 sccm of Ar, 10 μbar, SENTECH SI 200 plasma system). Finally, the resist was removed in oxygen plasma for 1 min (200 cm^3^ min^−1^ O_2_, 100 W, PVA TePla 300) followed by an acetone bath for 5 min.

### Design, folding and purification of DNA origami structures

All structures were designed using caDNAno v.0.2^28^. For the cryo-EM reconstruction of the turbine part, all structures were designed with a compact beam on top of each turbine structure (Supplementary Figs. 6 and 7) and designed only using a 7,560-base-long scaffold. The folding reaction mixtures contained a final scaffold concentration of 50 nM and oligonucleotide strands (IDT) of 500 nM. The folding reaction buffer contained 5 mM Tris, 1 mM EDTA, 5 mM NaCl and 20 mM MgCl_2_. The folding solutions were thermally annealed using TETRAD (MJ Research, now Bio-Rad) thermal cycling devices. The reactions were left at 65 °C for 15 min and then subsequently subjected to a thermal annealing ramp from 60 °C to 20 °C (1 °C h^−1^). The folded structures were purified from excess oligonucleotides by physical extraction from agarose gels and stored at room temperature until further usage. The list of oligonucleotides can be found in Supplementary Information.

The turbine structure with a long DNA bundle as load was designed using a scaffold of 8,064 bases and a scaffold of 9,072 bases. The folding reaction mixtures contained a final scaffold concentration of 10 nM plus oligonucleotide strands (IDT) of 100 nM each. The folding reaction buffer contained 5 mM Tris, 1 mM EDTA, 5 mM NaCl and 15 mM MgCl_2_ for the left-handed and right-handed versions or 20 mM MgCl_2_ for the achiral version of the turbine. The folding reaction mixtures were thermally annealed using TETRAD (MJ Research) thermal cycling devices. The reactions were left at 65 °C for 15 min and then subjected to a thermal annealing ramp from 60 °C to 20 °C (1 °C h^−1^). The folded structures were purified from excess oligonucleotides by polyethylene glycol precipitation and stored at room temperature until further usage. Details of all the procedures can be found in ref. ^29^.

### Cryo-EM sample preparation, image acquisition and processing

Grid preparation, image acquisition and data processing were largely performed as reported previously^30^. The sample was applied to a glow-discharged C-Flat 1.2/1.3 4C thick grid (Protochips) and vitrified using a Vitrobot mark IV (FEI, now Thermo Scientific) at a temperature of 22 °C, a humidity of 100%, 0 s wait time, 2 s blot time, −1 blot force (arbitrary device units) and 0 s drain time. Micrograph videos with 10 frames were collected for the right-handed and left-handed versions (3,427 and 5,997 respectively) at a magnified pixel size of 2.28 Å and an accumulated dose of ~60 e Å^−^^2^ using the EPU software and a Falcon 3 detector (FEI) on a Cs-corrected (CEOS) 300 kV Titan Krios electron microscope (FEI). For the left-handed version, acquisition with a stage tilt of 20° was used to reduce the orientation bias of the particles.

Motion correction and contrast transfer function estimation of the micrographs were performed using the implementation in RELION 4.0 beta^31,32^ and CTFFIND4, respectively^33^. Particles were autopicked using TOPAZ^34^ and subjected to a selection process consisting of multiple rounds of 2D and 3D classification in RELION to remove falsely picked particles and damaged particles. Using an ab initio initial model, a refined 3D map was reconstructed from 97,054 and 71,992 particles for the right-handed and left-handed versions, respectively, followed by per-particle motion correction and dose weighting and 3D refinement (Supplementary Figs. 6 and 7). For a focused reconstruction of the turbine, multibody refinement^35^ was performed. The consensus map was divided into two parts containing the lever and the turbine using the eraser tool in UCSF Chimera^36^, and low-pass-filtered soft masks of the respective regions were created in RELION (Supplementary Figs. 8 and 9). After multibody refinement, a set of particles with the subtracted signal of the lever arm was calculated and subjected to another round of 3D refinement. The final maps were masked, sharpened and low-pass filtered using the estimated resolution based on the 0.143 Fourier shell correlation criterion. Atomic models were constructed using a cascaded relaxation protocol as described previously^30^ (Supplementary Fig. 11).

The dimensions of the turbines were measured in Fiji^37^ using orthographic projections of the maps created with ChimeraX^38^. For the twist measurement of the turbine versions, slices from the well resolved central parts were extracted from the cryo-EM density maps using atomic model fits at base-pair positions that are on the same plane in the design, with a spacing of 33 bp and 34 bp for the right- and the left-handed version, respectively (Supplementary Fig. 10). For each version, the slices were fitted into each other on the basis of maximum overlay using ChimeraX^38^ to determine the rotation angle. From the twist density, the diameter and the length of the helices, the outer blade angle with respect to the helical axes was calculated.

### Single-particle fluorescence imaging

Solid-state nanopore chips were oxygen-plasma cleaned before all the fluorescence experiments (100 W for 1 min, Plasma Prep III, SPI Supplies). Coverslips (VWR, no. 1.5) were cleaned by ultrasonication sequentially in acetone, isopropanol, water, 1 M KOH solution and deionized water (Milli-Q) for 30 min each. The cleaned coverslips were then blow-dried thoroughly with compressed nitrogen. The nanopore chip was glued into the PDMS (SYLGARD 184 silicone elastomer) flow cell using a two-component silicone rubber (Ecoflex 5, Smooth-ON), then the PDMS flow cell was bonded to the cleaned coverslip after oxygen-plasma treatment (50 W, 50 mbar for 30 s) and post-bake at 120 °C for 30 min. After assembly, the whole device was again treated with oxygen plasma (50 W, 50 mbar) for 4 min before embedding a pair of Ag/AgCl electrodes, one in each side of the reservoir, and flushing in deionized water to wet the channels. This is essential for increasing the hydrophilicity of the membrane and ensuring a negatively charged silicon nitride surface. The PDMS nanopore devices were always assembled shortly before each experiment and never reused.

The nanopore chip was then imaged using an epifluorescence microscope with a ×60 water immersion objective (Olympus UPlanSApo, numerical aperture 1.20) and a fast scientific complementary metal–oxide–semiconductor camera (Prime BSI, Teledmy Photometrics). The camera field of view was reduced as needed to achieve high frame rates (typically around 200 pixels × 200 pixels). To image Cy3-labelled DNA turbines, a 561 nm laser (Stradus, Vortran Laser Technology) was used to excite the fluorophores. The typical exposure time of the experiments was 5 ms, which led to a frame rate of around 190–200 fps. To simplify the data analysis, a fixed frame rate value (200 fps) is used. Before imaging, the imaging buffer (50 mM Tris-HCl pH 7.5; 50 mM NaCl unless otherwise stated, 5 mM MgCl_2_; 1 mM dithiothreitol, 5% (w/v) d-dextrose, 2 mM Trolox, 40 μg ml^−1^ glucose oxidase, 17 μg ml^−1^ catalase; 0.05% TWEEN 20) was placed into the reservoirs on either side of the silicon nitride membrane.

### Driving DNA turbines using transmembrane ion gradients

An imaging buffer with the same salt concentration (50 mM NaCl) was flushed into the flow cell first, with DNA turbines on the *cis* side of the membrane. Subsequently, an imaging buffer containing a higher NaCl concentration was flushed into the *trans* side of the membrane. With single-particle fluorescence microscopy, the docking and rotation of the DNA turbines could be observed and recorded. To release the turbines from the nanopore, we either inserted a pair of temporary electrodes into the inlet and outlet of the flow channels and released the turbines electrically, or we flushed in the same (lower-concentration) buffer as the *cis* side. Because of the photobleaching and accumulation of the DNA turbines near nanopore arrays, we chose 40 s as a typical observation duration. Examples of longer recordings are shown in Supplementary Fig. 23.

### Driving DNA turbines using transmembrane voltages

In contrast to the salt-gradient-driven mode, a pair of electrodes was embedded into the flow cells. We used a custom-built circuit to apply voltages^39^. The output voltage was controlled by a custom LabVIEW program. The electrodes embedded in the two reservoirs were connected to the circuit. The DNA origami turbines were placed into the electrically grounded side (*cis* side) of the flow cell with a typical concentration of 1 pM. After applying the voltage, the DNA turbines were docked onto the nanopores under a 100 mV bias voltage (unless otherwise stated) across the membrane. The turbines could be easily released from the nanopore array by flipping the voltage polarity and then setting it to 0 mV for several seconds to allow the imaged turbines to diffuse away from the capture region. To avoid overcrowding of DNA turbines near the nanopore array, which increased the fluorescence background fluctuation, we typically imaged the turbines before the array was fully filled, and subsequently released them from the nanopore. Then a new group of turbines could be captured and docked again by applying a positive bias.

### Fluorescence microscopy data analysis

For image processing, first a single-molecule localization was carried out using Fiji (ImageJ^37^) with the ThunderSTORM plugin^40^ for all frames in the acquired image sequences. A wavelet filter (B-spline) and an integrated Gaussian method were used for the localizations. Then the results were filtered on the basis of their quality (uncertainty < 50 nm) and the local density (filter of 15 particles in every 50 nm among all localized data points in the sequence) to rule out free-diffusing (non-captured) turbines. Next, the single-molecule localization results were analysed using a custom MATLAB script (Code availability). In brief, all coordinates of localized particle positions were clustered on the basis of their Euclidean distance for each turbine. When localized particle positions were deduced in a video, a circle was fitted to the data to obtain the centre and a radius, which subsequently was used for calculating the angular position of the fluorophores in each frame. Next, we determined if the fluorophores occupy spatial states that can be fitted to a circular path. We did this by comparing the point density of the coordinates within an annulus around the fitted circular perimeter (±1 nm) with the density of points around the centre (with a radius *r*, so that the area of this central circle is equal to that of the ring region). If the point density within the annulus was higher, then this data group would be kept in the statistics, else it would be considered an invalid trajectory and discarded. Finally, the script calculated all necessary motion properties of the turbine, including its cumulative angular displacements, MSD, angular velocity and torque. The angular velocity *ω*_d_ was determined by fitting MSD = *ω*_d_^2^*t*^2^ + 2*D*_r_*t* to the MSD curve of each turbine, where *t* is the lag time and *D*_r_ is the rotational diffusion coefficient (also as a fitting parameter). The estimation of the torque is discussed in Supplementary Section 1.

### MD simulations

All MD simulations were performed using the NAMD program^41^, CHARMM36 parameters for DNA, water and ions^42^ with CUFIX^43^ corrections, periodic boundary conditions and the TIP3P model of water^44^. The long-range electrostatic interactions were computed using the particle-mesh Ewald scheme over a grid with 1 Å spacing^45^. Van der Waals and short-range electrostatic forces were evaluated using the 10–12 Å smooth cutoff scheme. Hydrogen mass repartitioning^46^ and the SHAKE^47^ and SETTLE^48^ algorithms were used, enabling a 4 fs integration time step. The full electrostatics were calculated every two-time step. Except where specified, a Langevin thermostat with a 0.1 ps^−1^ damping coefficient maintained a temperature of 295 K in all simulations. Coordinates were recorded every 2,500 steps.

Atomistic models of the entire turbine were assembled from the caDNAno^28^ design file using a custom mrdna script^49^. In addition to neutralizing Mg^2+^, 10 mM Mg^2+^ hexahydrate was placed adjacent to the DNA according to a previously described protocol^43^. Water and monovalent ions were added to the system using the solvate and autoionize plugins for VMD^50^, with the solvent box cut to form a hexagonal prism. For each salt condition, a 4 ns simulation was performed with a Nosé–Hoover Langevin piston barostat^51,52^ set to maintain a target pressure of 1 bar, allowing the equilibrium volume of the system to be determined. The resulting system dimensions were used in constant-volume simulations to equilibrate the turbine with harmonic position restraints holding the phosphorus atoms to their initial coordinates during the first 7 ns of the simulation (*k*_spring_ = 1 kcal mol^–1^ Å^–2^ for *t* < 5 ns; 0.1 for 5 ns < *t* < 7 ns). After 30 ns of equilibration for the 50 mM and 75 ns for the 3 M system, a snapshot of the configuration was used to initialize subsequent simulations with either an electric field or pressure gradient applied to drive the turbine. Additional equilibration was performed for the 50 mM NaCl system for another 128 ns to initialize the ‘Alternate conf.’ system (Supplementary Fig. 24).

The conformation of the turbine at the end of equilibration was used to determine the rest positions of several harmonic collective variable (colvar)^53^ potentials, including a spring restraining the centre of mass of every third phosphorus atom (*k*_spring_ = 500 kcal mol^–1^ Å^–2^); a spring restraining the root-mean-square deviation (RMSD) of these phosphorus atoms with respect to the post-equilibration configuration (*k*_spring_ = 1,000 kcal mol^–1^ Å^–2^; resting RMSD = 0), after optimal rigid body transformations so that the potential does not apply a net torque or force; and a pair of centre of mass harmonic restraints applied to 16-bp-long sections of the central six-helix bundle (*k*_spring_ = 50 kcal mol^–1^ Å^–2^), placed near either the end of the shaft to prevent the turbine from tilting. With these colvars preventing translation, conformational fluctuations, or tilting of the turbine, an electric field was applied by placing a constant force on each atom with a magnitude proportional to the charge of the atom. Similarly, a pressure gradient was achieved by placing a small force on every water molecule of the system. Finally, in simulations where the torque was measured, an additional spin angle colvar (*k*_spring_ = 100 kcal mol^–1^ °^–2^) prevented rotation of the turbine and reported the torque.

Simulation systems were prepared to study the forces on and flows around a DNA helix mimicking the DNA in the turbine blade. The 21 bp helix was made effectively infinite by connecting the ends of each strand across the periodic boundary. Solvent (neutralizing Na^+^, 100 mM and 3 M NaCl; no Mg^2+^) was added around the helix. Systems were equilibrated for 5–50 ns with the DNA phosphorus atoms harmonically restrained (*k*_spring_ = 0.2 kcal mol^–1^ Å^–2^). Mobility measurements were performed using a field of 5 mV nm^−1^ or a hydrostatic pressure of ~1.3 bar nm^−1^ parallel or transverse to the helical axis, with each condition employing four replicate simulations lasting a total of 400 (neutralizing Na^+^) to 4,000 ns (3 M). Except where otherwise specified, a 100 mV nm^−1^ electric field was applied to the system at a 35° angle with respect to the DNA while a centre of mass colvar restrained the DNA (*k*_spring_ = 500 kcal mol^–1^ Å^–2^), an RMSD colvar retained an idealized DNA configuration (*k*_spring_ = 100 kcal mol^–1^ Å^–2^) and a spin angle colvar (*k*_spring_ = 100 kcal mol^–1^ °^–2^) prevented rotation of the DNA and reported on the torque. Eight replicate systems were employed during simulations lasting a total of 1,040 ns (100 mM) or 2,055 ns (3 M). The flow and concentration of ions and water oxygen atoms were analysed by binning the system into ~1 Å voxels, counting the flux through and concentration in each voxel using a centred finite-difference approximation for the flux. The difference in concentration between sodium and chloride ions provided the net local charge density of the fluid around the DNA in each case. Multiplying this charge by the electric field provided the solvent force. In subsequent simulations, the 3D map of the solvent force was used to apply a position-dependent force to each water oxygen atom using the TclBC feature of NAMD and employing the approximation that the density of water oxygen atoms is uniformly 33 nm^−^^3^. Again, eight replicate systems were employed for simulations lasting a total of 480 and 570 ns for 100 mM and 3 M conditions, respectively.

### Statistics and reproducibility

No statistical method was used to predetermine the sample size.

## Online content

Any methods, additional references, Nature Portfolio reporting summaries, source data, extended data, supplementary information, acknowledgements, peer review information; details of author contributions and competing interests; and statements of data and code availability are available at 10.1038/s41565-023-01527-8.

## Supplementary information

Supplementary Information Supplementary text, Figs. 1–26 and video captions.

Supplementary Table 1 DNA origami sequences for the turbines.

Supplementary Video 1 MD simulation of the right-handed DNA turbine rotation in 100 mM NaCl under electric field, as shown in Fig. 4.

Supplementary Video 2 MD simulation of the right-handed DNA turbine rotation in 3 M NaCl under electric field, as shown in Fig. 4.

## Source data

Source Data Fig. 2 Source Data Fig. 2.

Source Data Fig. 3 Source Data Fig. 3.

Source Data Fig. 4 Source Data Fig. 4.

## Data Availability

The electron density maps of the left- and right-handed turbines are available in the Electron Microscopy Data Bank (EMDB) as entries EMD-17600 and EMD-17606, respectively. Fluorescence microscopy and nanopore experimental data are available at 10.5281/zenodo.8091178. Simulation trajectory data are available at 10.13012/B2IDB-3458097_V1. Source data are provided with this paper.

## Code availability

The corresponding MATLAB scripts for data processing and producing the final figures are available at 10.5281/zenodo.8091178. Scripts related to simulation set-up and analysis are available at 10.13012/B2IDB-3458097_V1.
