# Supplementary material for: A DNA turbine powered by a transmembrane potential across a nanopore
Source: Nat Nanotechnol. 2023 Oct 26;19(3):338–44. doi: 10.1038/s41565-023-01527-8 (PMC10950783; doi:10.1038/s41565-023-01527-8)
Supplement: Supplementary file 1 — Supplementary text, Figs. 1–26 and video captions. [file 41565_2023_1527_MOESM1_ESM.pdf]

nature nanotechnology

Article

https://doi.org/10.1038/s41565-023-01527-8

A DNA turbine powered by a transmembrane potential across a nanopore

In the format provided by the authors and unedited

This PDF file includes:

Supplementary Text
Supplementary Figures. S1 to S26
Supplementary Movie Captions

Other Supplementary Materials for this manuscript include the following:

Supplementary Movie S1-S2: Simulated DNA turbine driven by voltage.
Supplementary Table S1: DNA origami sequences for the turbines.

Supplementary Text

1. Estimate the torque of the turbine from MSD curves.

We estimate the torque that the turbine exerts as follows. We adopt the following assumptions about the system: 1) We assume that the DNA bundle behaves as a passive load; 2) we simplify the bundle to a stiff cylindrical-shaped rod with dimensions of a 250 nm length and 12 nm diameter, i.e. considering only slightly longer than the middle reinforced part of the used DNA bundle to contribute to the load; and 3) we neglect any surface effects resulting from the close proximity of the membrane to the DNA bundle.

For purely rotational diffusion about a single axis, the mean-square angular deviation in time *t* is

$$\langle\theta^2\rangle = 2D_r t, \quad (1)$$

where θ is the measured rotation angle (in rad), D_r is the rotational diffusion coefficient (in $\mathrm{rad}^2/\mathrm{s}$). Additionally, ignoring diffusion, the angular drift velocity $\omega_d = (d\theta/dt)_{drift}$ in response to an external torque Γ_θ results in

$$\langle\theta^2\rangle = \omega_d^2 t^2,$$

with

$$\omega_d = \frac{\Gamma_\theta}{f_r}, \quad (2)$$

where f_r is the frictional drag coefficient. The relationship between the rotational diffusion coefficient and the rotational frictional drag coefficient is given by Einstein–Smoluchowski relation:

$$D_r = \frac{k_B T}{f_r}, \quad (3)$$

where k_B is the Boltzmann constant and T is temperature. These relationships are in complete analogy to translational diffusion. The hydrodynamic drag of the DNA rod is estimated by treating it as a cylinder of length 250 nm and 6 nm radius rotating about its middle point along the long axis, for which f_r can be estimated as [1]

$$f_r = \frac{\pi\eta L^3}{3(\ln p + \delta_T)}, \quad (4)$$

where η is the solvent viscosity, L is the cylinder length, δ_T is an end-effect correction, and

$$p = \frac{L}{2R}, \quad (5)$$

where R is the cylinder radius. For our system, we have $\eta = 10^{-3}\mathrm{Pa}\cdot\mathrm{s}, L = 250\ \mathrm{nm}, R = 6\ \mathrm{nm}, p = \frac{250}{2\times6} \approx 20.8$, and we use $\delta_T = -0.616$ as an approximate end-effect correction [1].

Now the contributions to the angular displacement of in response to both diffusion and external torque add linearly and we can fit the model

$$\langle\theta^2\rangle = a_1 t^2 + a_2 t = \omega_d^2\, t^2 + 2D_r t, \quad (6)$$

to our data for $\theta(t)$, where ω_d is the angular drift velocity, a_1 and a_2 are the two fitting parameters, as

$$a_1 = \left(\frac{\Gamma_\theta}{f_r}\right)^2, \tag{7}$$

From equation (1) we thus obtain the torque

$$\Gamma_\theta = f_r\sqrt{a_1}, \tag{8}$$

This was implemented into a MATLAB routine to estimate torques for every DNA turbine, and the results are shown in Fig. S19.

2. Continuum hydrodynamic model for the reversal of the rotational direction

Consider a high-aspect ratio rigid cylindrical DNA rod in a wide nanopore that is orientated at an angle of θ with respect to the Z axis, as shown in the schematic in Scheme S1.

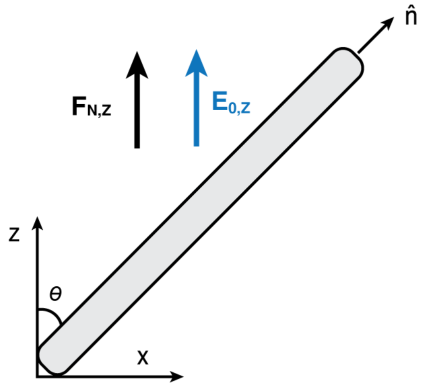

Scheme S1. A rigid cylindrical DNA rod.

The net motion of this single cylindrical DNA rod that is held in the applied E_z field in the nanopore is governed by the equation:

$$\boldsymbol{v} = \left[M_{el,\parallel}\,\hat{\boldsymbol{n}}\hat{\boldsymbol{n}} + M_{el,\perp}(\boldsymbol{I} - \hat{\boldsymbol{n}}\hat{\boldsymbol{n}})\right] \cdot \boldsymbol{E_Z} + \left[M_{h,\parallel}\,\hat{\boldsymbol{n}}\hat{\boldsymbol{n}} + M_{h,\perp}(\boldsymbol{I} - \hat{\boldsymbol{n}}\hat{\boldsymbol{n}})\right] \cdot \boldsymbol{F_{N,Z}}\,, \tag{1}$$

$$v_z = \left[M_{el,\parallel}\cos^2\theta + M_{el,\perp}\sin^2\theta\right] \cdot E_Z + \left[M_{h,\parallel}\cos^2\theta + M_{h,\perp}\sin^2\theta\right] \cdot F_{N,Z} \tag{2}$$

$$v_x = \sin\theta\cos\theta\left[(M_{el,\parallel} - M_{el,\perp})\,E_Z + (M_{h,\parallel} - M_{h,\perp})\,F_{N,Z}\right], \tag{3}$$

where $v_z = 0$ because the rod is vertically held in position. Hence it follows that

$$F_{N,Z} = -\left(\frac{M_{el,\parallel}\cos^2\theta + M_{el,\perp}\sin^2\theta}{M_{h,\parallel}\cos^2\theta + M_{h,\perp}\sin^2\theta}\right) \cdot E_Z \tag{4}$$

Combining (3) and (4) yields and expression for v_x as follows

$$v_x = \sin\theta\cos\theta\left[(M_{el,\parallel} - M_{el,\perp}) - (M_{h,\parallel} - M_{h,\perp})\left(\frac{M_{el,\parallel}\cos^2\theta + M_{el,\perp}\sin^2\theta}{M_{h,\parallel}\cos^2\theta + M_{h,\perp}\sin^2\theta}\right)\right] \cdot E_z \tag{5}$$

This equation (5) can be rewritten as

$$v_x = \frac{1}{2} M_{el,\parallel} \sin 2\theta \cdot \sigma \cdot E_z, \tag{6}$$

Where

$$\sigma = \frac{\left(\frac{M_{h,\perp}}{M_{h,\parallel}} - \frac{M_{el,\perp}}{M_{el,\parallel}}\right)}{\cos^2\theta + \left(\frac{M_{h,\perp}}{M_{h,\parallel}}\right)\sin^2\theta} \tag{7}$$

Notably, $M_{h,\perp}/M_{h,\parallel} = 0.5$ for a thin long rod, while $M_{el,\perp}/M_{el,\parallel}$ depends on the salt concentration and ranges between 0 and 1. Specifically, $M_{el,\perp}/M_{el,\parallel}$ depends on the Dukhin number $Du = \frac{\sigma\, l_B}{\kappa^2 b} = \frac{\sigma}{4\pi C_0 b}$, where σ is the surface charge density, 1/κ is the Debye length, and l_B is Bjerrum length , C_0 is the salt concentration, and b is the DNA radius. For DNA, $Du = \frac{0.2}{[C_0]}$. Using values from Ref. 2, we can estimate $M_{el,\perp}/M_{el,\parallel}$ for various salt concentrations as follows:

Du	C_0	$\frac{M_{el,\perp}}{M_{el,\parallel}}$
6.43	30 mM	0.14
0.569	350 mM	0.44
0.0645	3 M	0.84

Based on these numbers, we expect σ to change sign somewhere between 500 mM and 1 M, which indeed is the salt range where the turbine experiments show a sign reversal of the rotations.

An alternative calculation that is similar to the MD simulations is to add a force in the x direction to equation (1) and demand $v_x = 0$. Equation (2) & (3) will then be modified as

$$v_z = \left[M_{el,\parallel}\cos^2\theta + M_{el,\perp}\sin^2\theta\right] \cdot E_Z + \left[M_{h,\parallel}\cos^2\theta + M_{h,\perp}\sin^2\theta\right] \cdot F_{N,Z} + \left[(M_{h,\parallel} - M_{h,\perp})\sin\theta\cos\theta\right] F_x \tag{8}$$

$$v_x = \left[(M_{el,\parallel} - M_{el,\perp})\sin\theta\cos\theta\right] E_Z + \left[(M_{h,\parallel} - M_{h,\perp})\sin\theta\cos\theta\right] F_{N,Z} + \left[M_{h,\parallel}\sin^2\theta + M_{h,\perp}\cos^2\theta\right] F_x \tag{9}$$

Setting $v_z = 0$ and $v_x = 0$, we can eliminate the $F_{N,Z}$ between (8) and (9), and solve for F_x as a function of E_Z. These yields:

$$F_x = -\frac{M_{el,\parallel}}{M_{h,\perp}}\frac{1}{2}\sin 2\theta\left(\frac{M_{h,\perp}}{M_{h,\parallel}} - \frac{M_{el,\perp}}{M_{el,\parallel}}\right) \tag{10}$$

This shows that the sign of F_x is controlled by the same competition between the mobility (anisotropy) ratios. Moreover, we observe that $F_x < 0$ at lower salt concentration and $F_x > 0$ at higher salt concentration (see discussion above), in agreement with the MD simulations results in Fig 4i of the main text.

Supplementary Figures

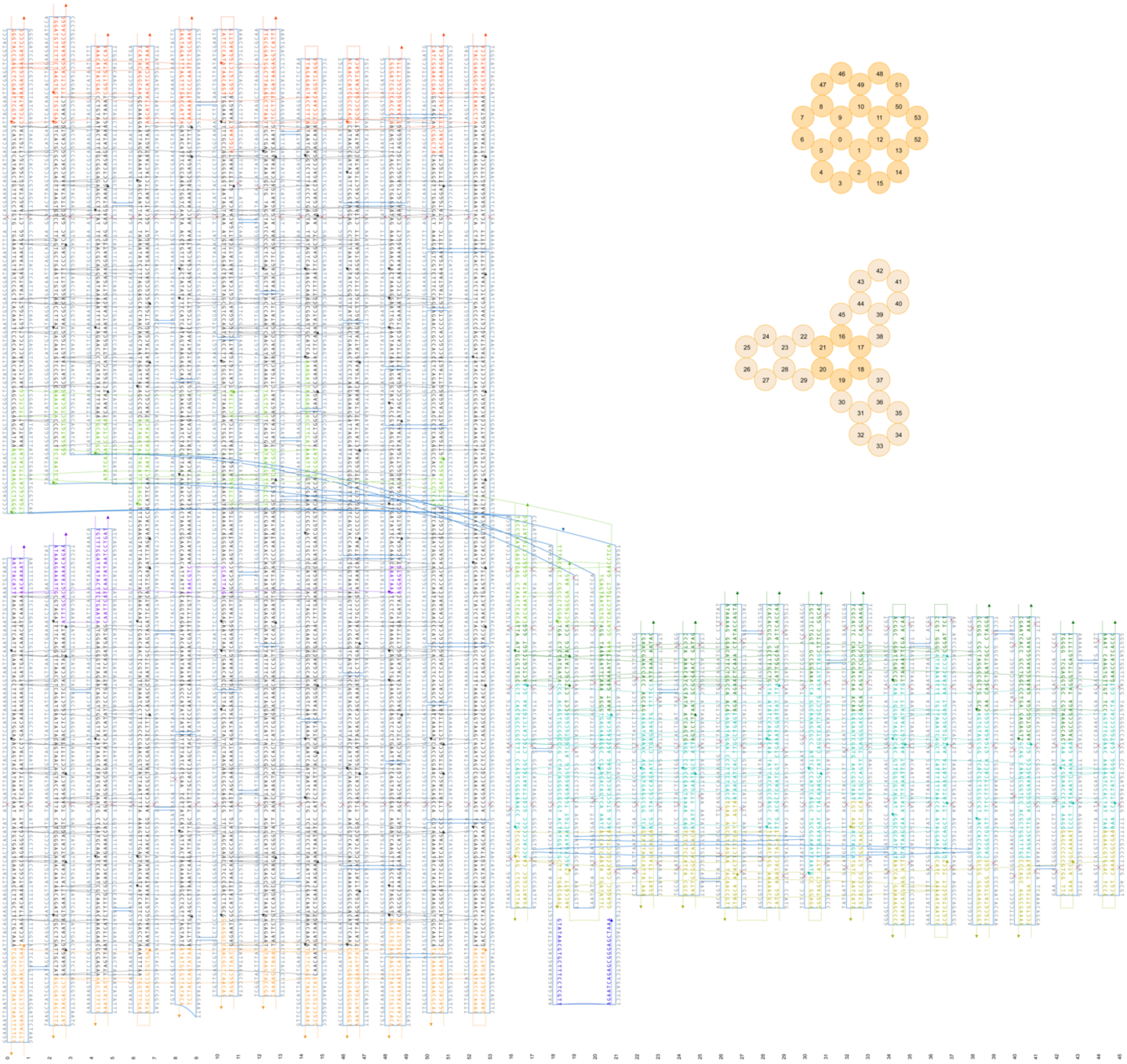

Figure S1. Design diagram of the left-handed turbine.
This design was created with caDNAno *(28)*.

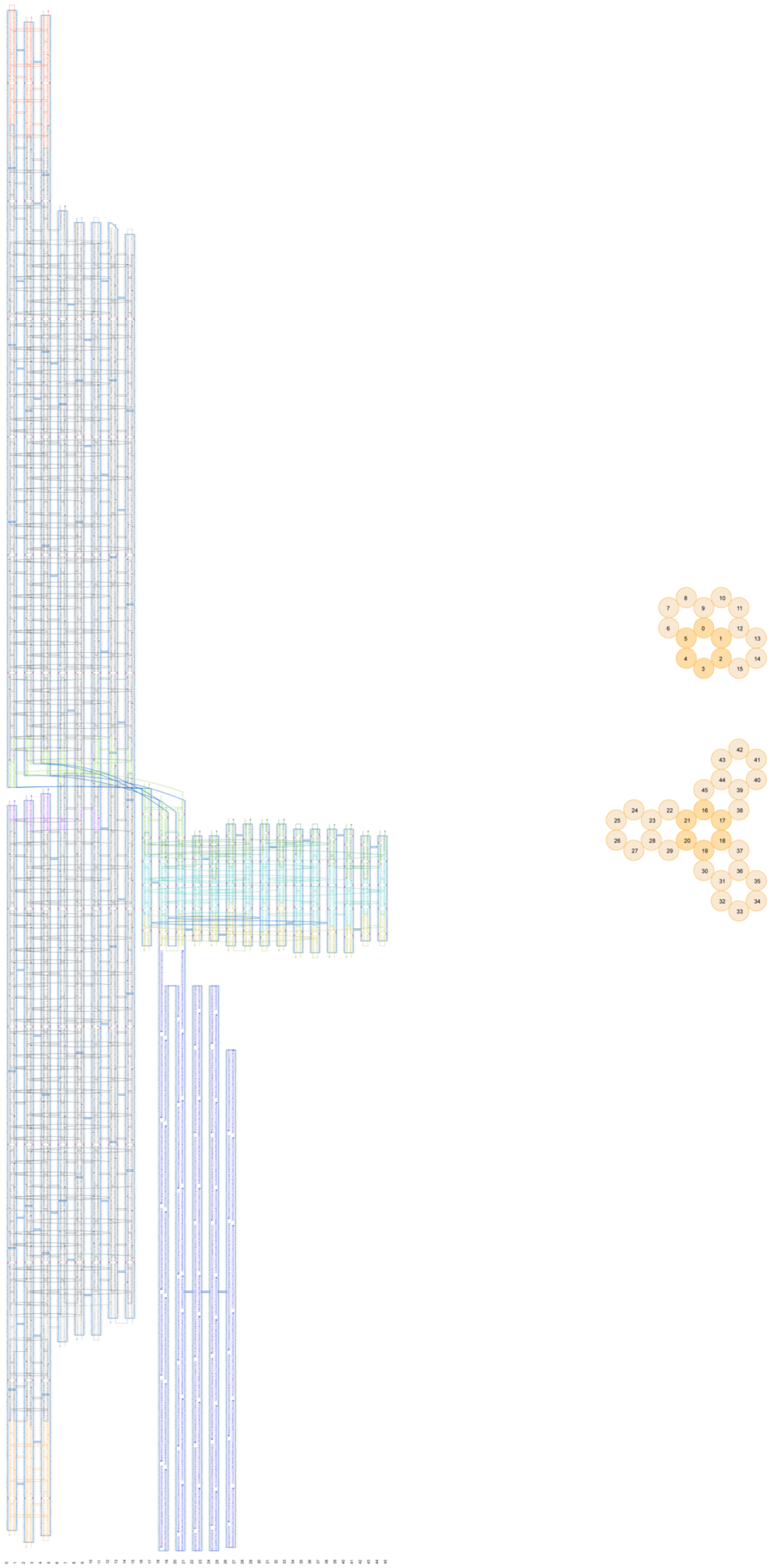

Figure S2. Design diagram of the left-handed turbine including load.
This design was created with caDNAno *(28)*.

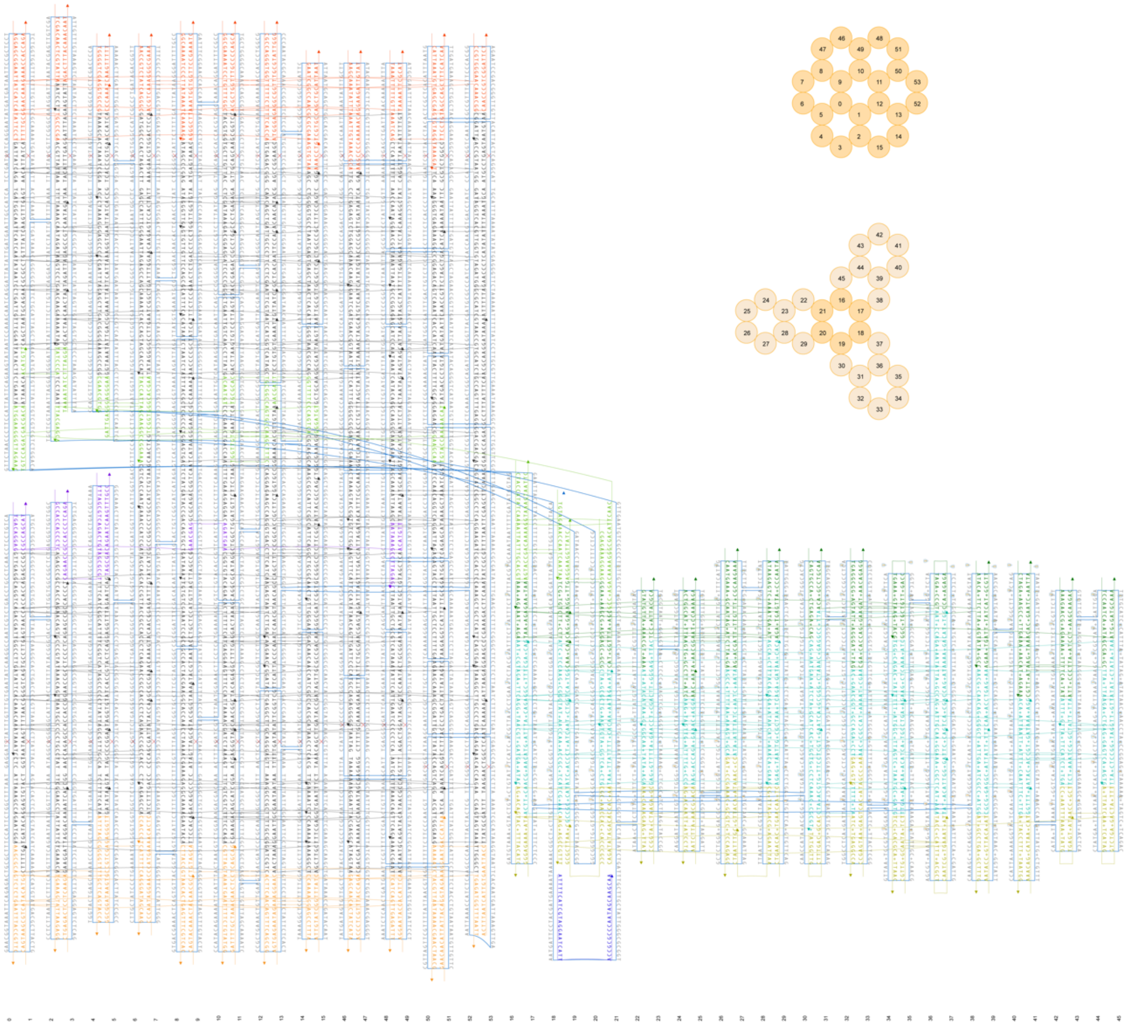

Figure S3. Design diagram of the right-handed turbine.
This design was created with caDNAno *(28)*.

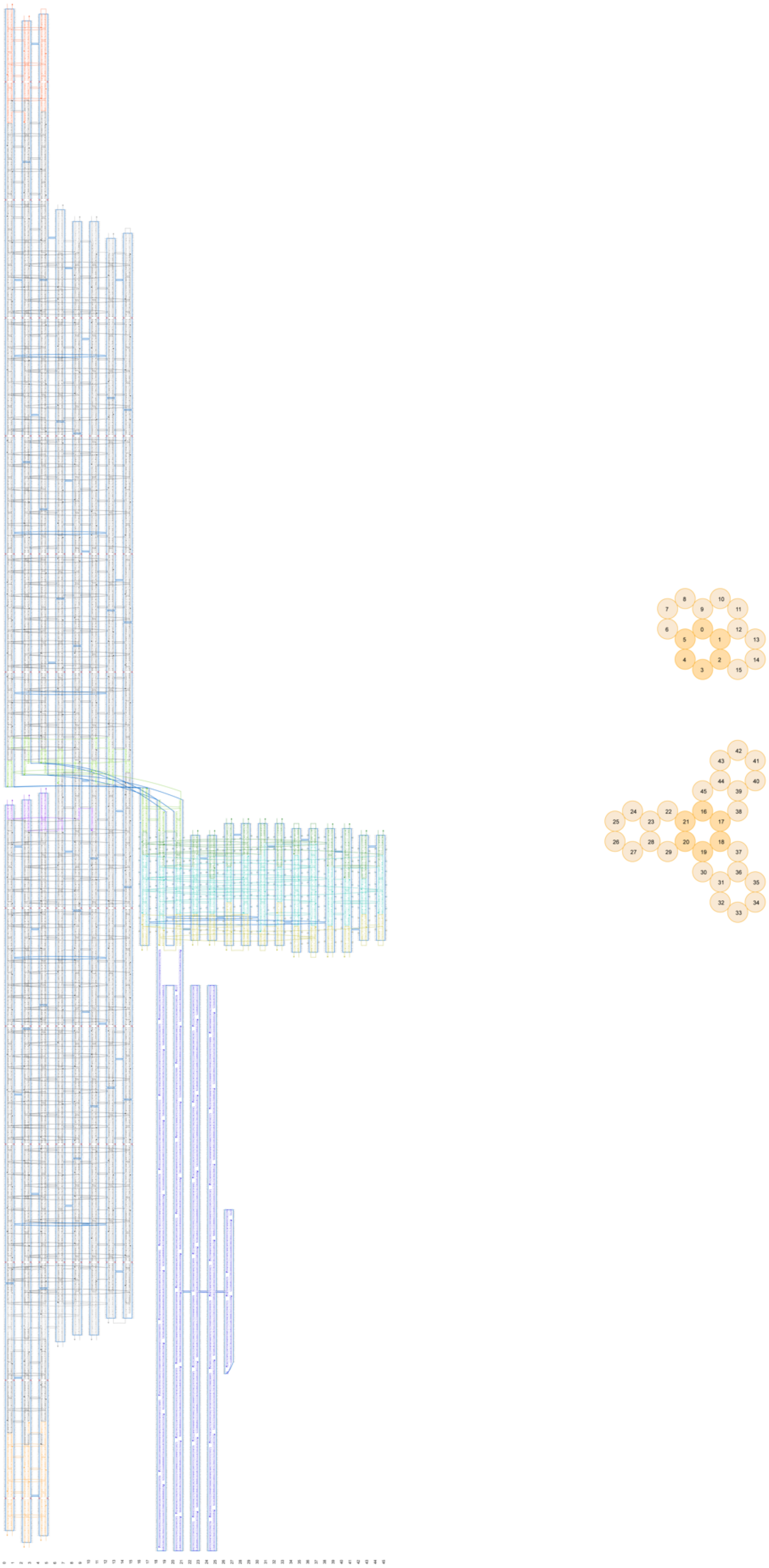

Figure S4. Design diagram of the right-handed turbine including load.
This design was created with caDNAno *(28)*.

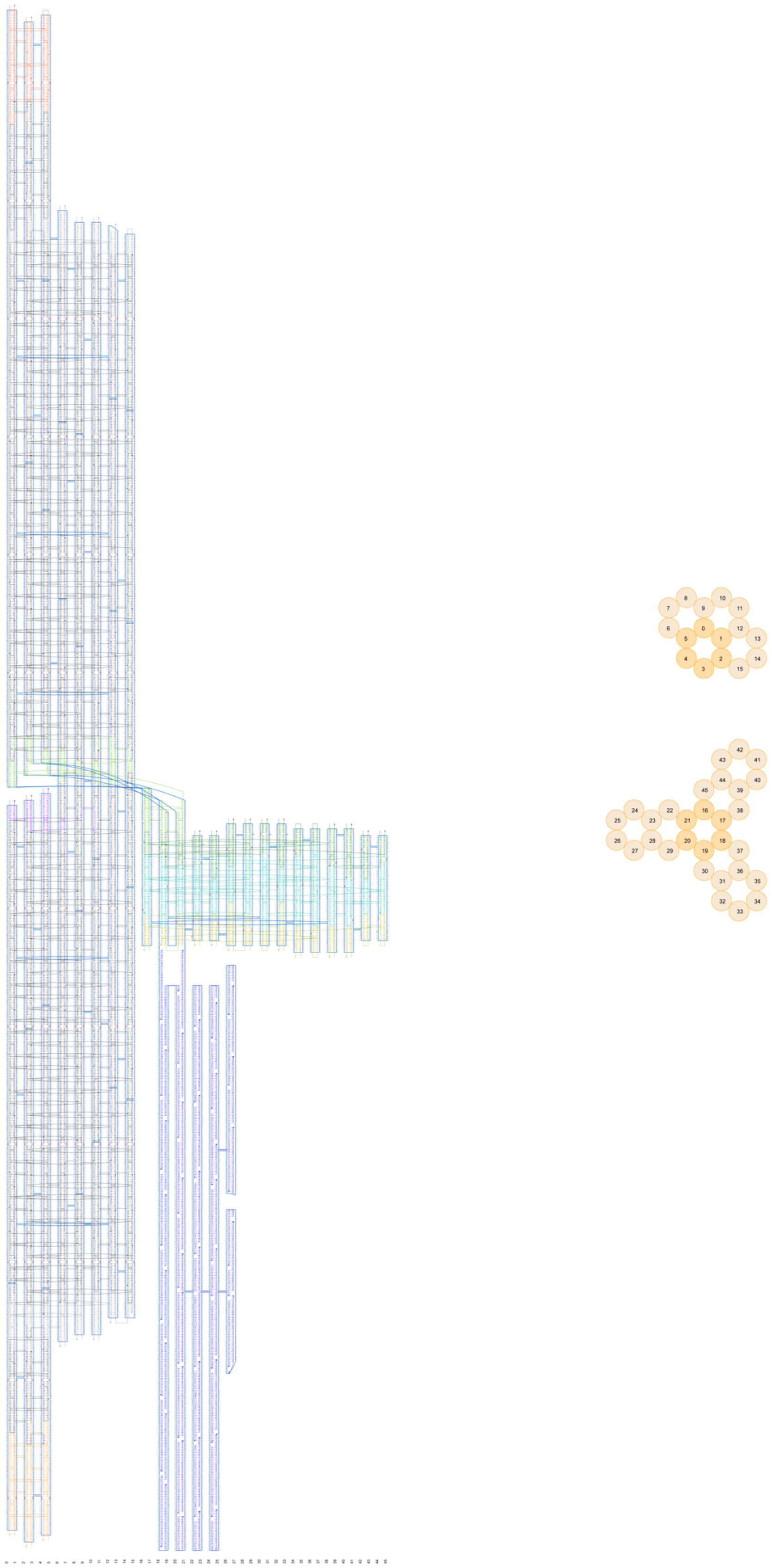

Figure S5. Design diagram of the achiral turbine including load.
This design was created with caDNAno *(28)*.

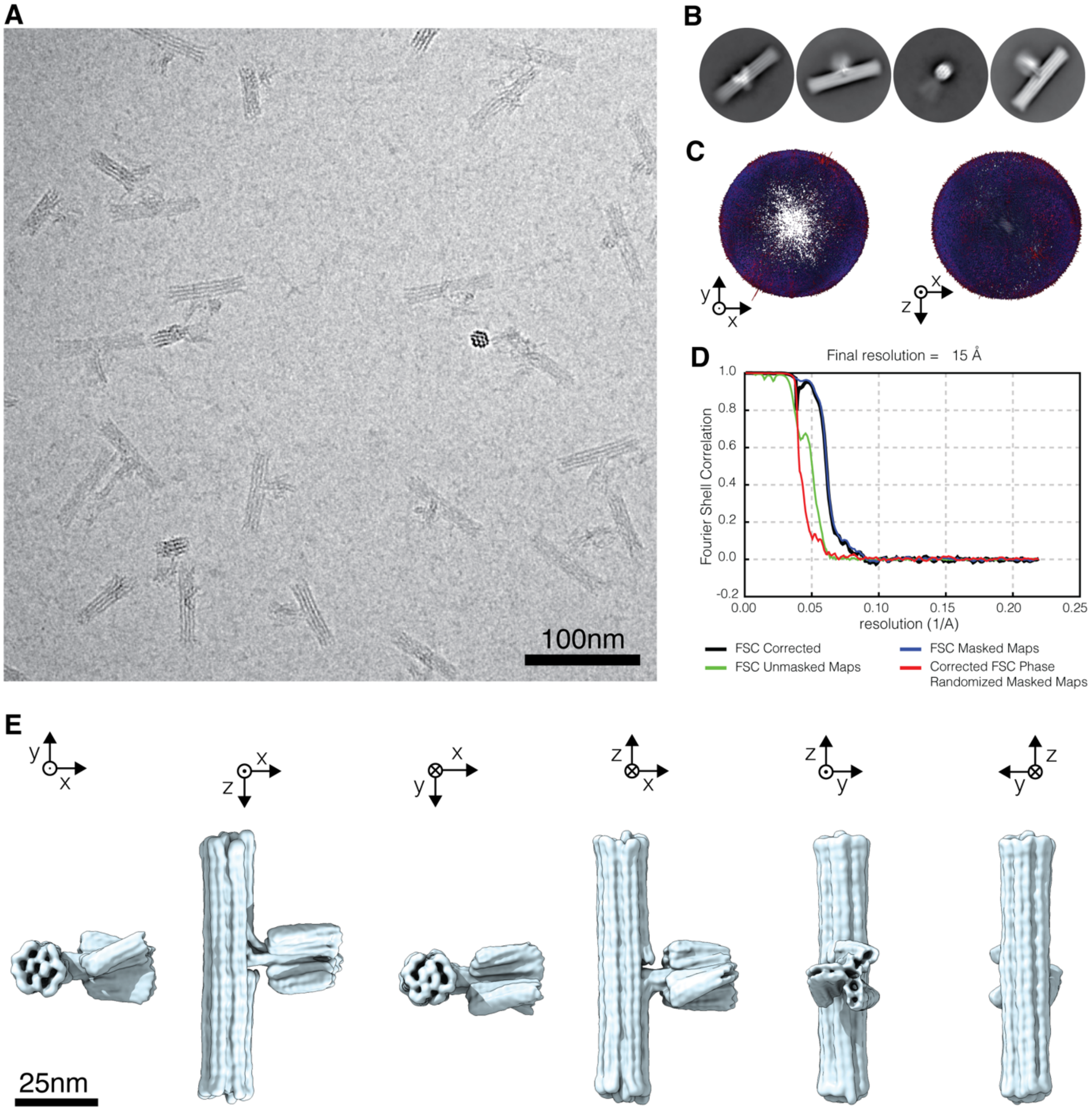

Figure S6. Cryo-EM map determination for the left-handed turbine variant.
(A) Exemplary micrograph of a total of 5,997 movie stacks. Scale bar is 100 nm. **(B)** Representative 2D class averages. **(C)** Histogram representing the orientational distribution of particles. **(D)** Fourier Shell Correlation (FSC) plot. **(E)** Six different views of the low-pass filtered electron density map. Scale bar is 25 nm.

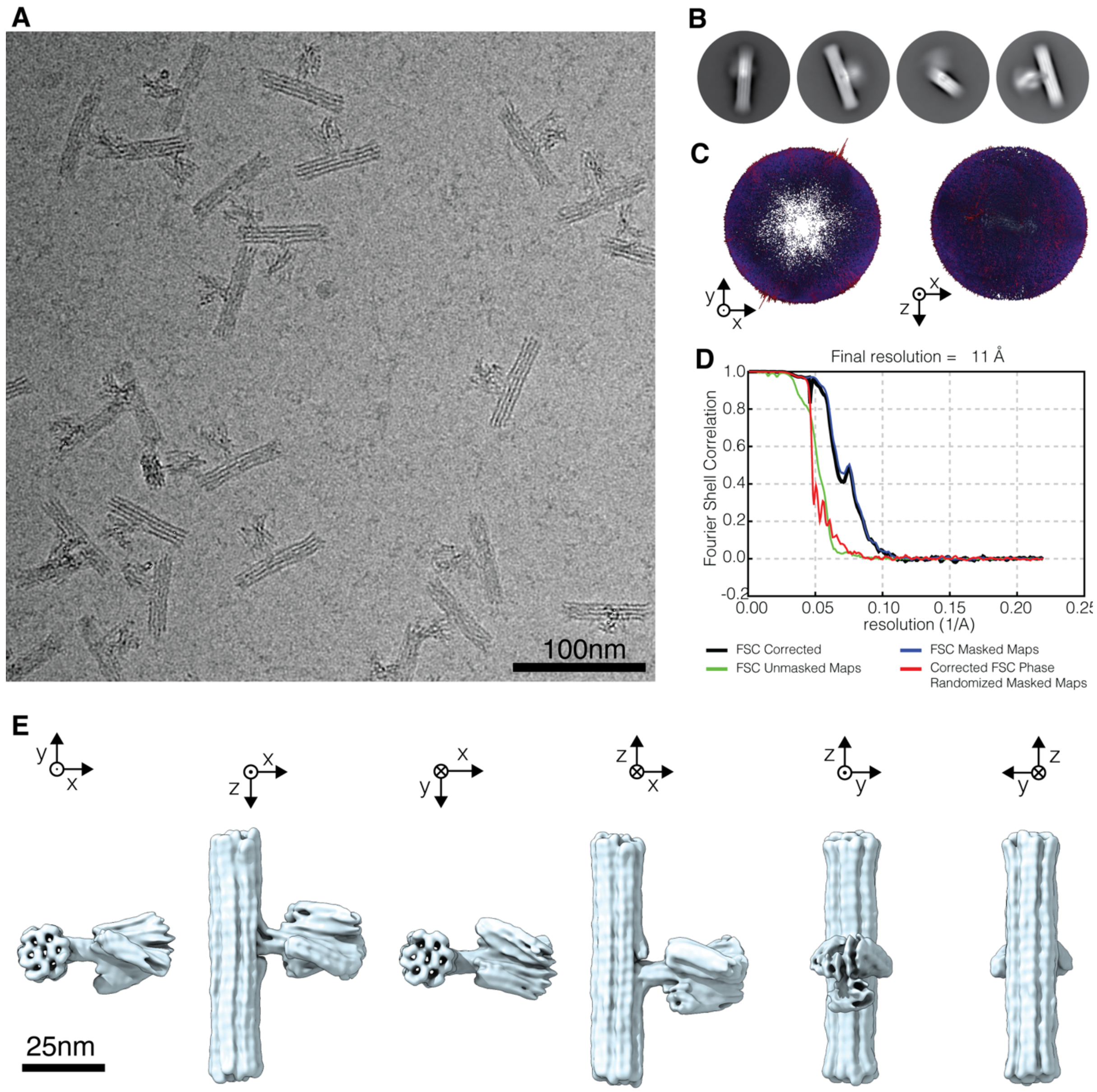

Figure S7. Cryo-EM map determination for the right-handed turbine variant.
(A) Exemplary micrograph of a total of 3427 movie stacks. Scale bar is 100 nm. **(B)** Representative 2D class averages. **(C)** Histogram representing the orientational distribution of particles. **(D)** Fourier Shell Correlation (FSC) plot. **(E)** Six different views of the low-pass filtered electron density map. Scale bar is 25 nm.

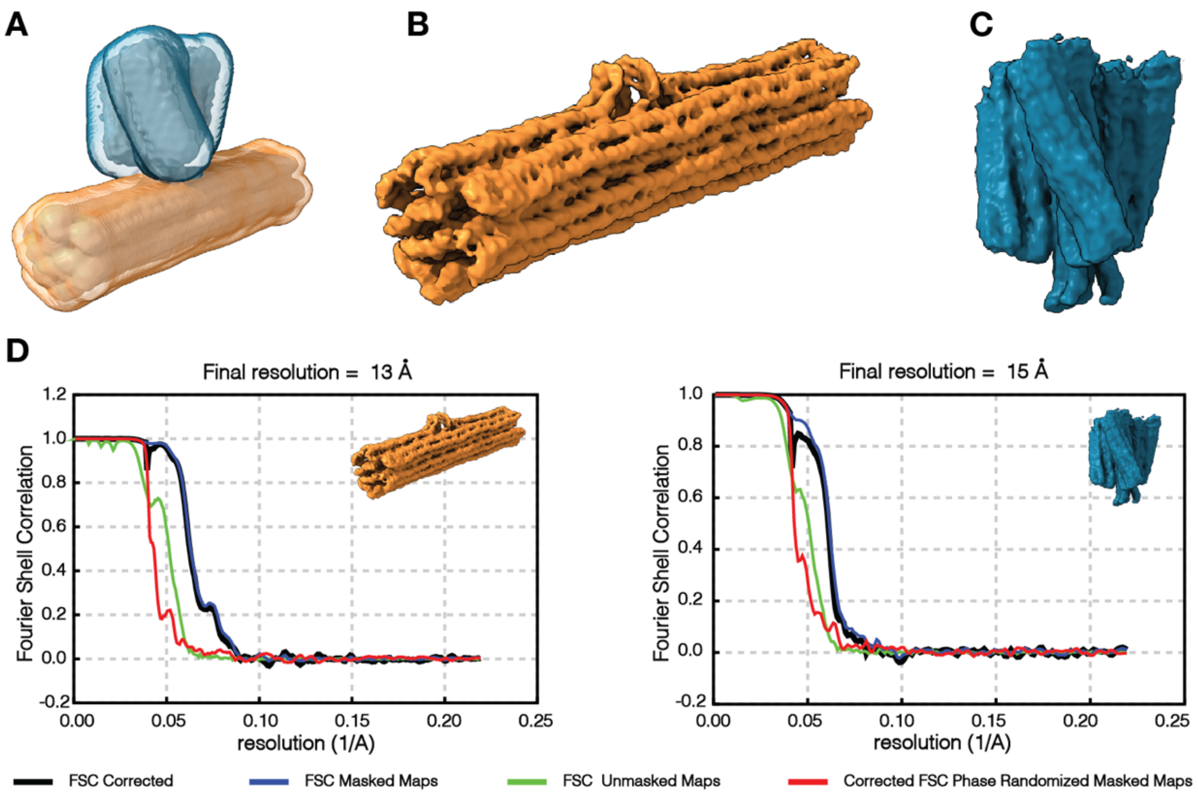

Figure S8. Multibody (MB) refinement-based reconstruction of the left-handed turbine variant.
(A) Masks of the rotor (blue transparent) and the lever (orange transparent) used for MB refinement. The map of the consensus refinement of the entire object is shown as a reference (gray). **(B)** Post-processed map of the MB-refined lever. **(C)** Post-processed map of the turbine reconstructed from a MB-refinement based set of partially-signal-subtracted particles. **(D)** FSC curves of the lever (left) and the turbine (right).

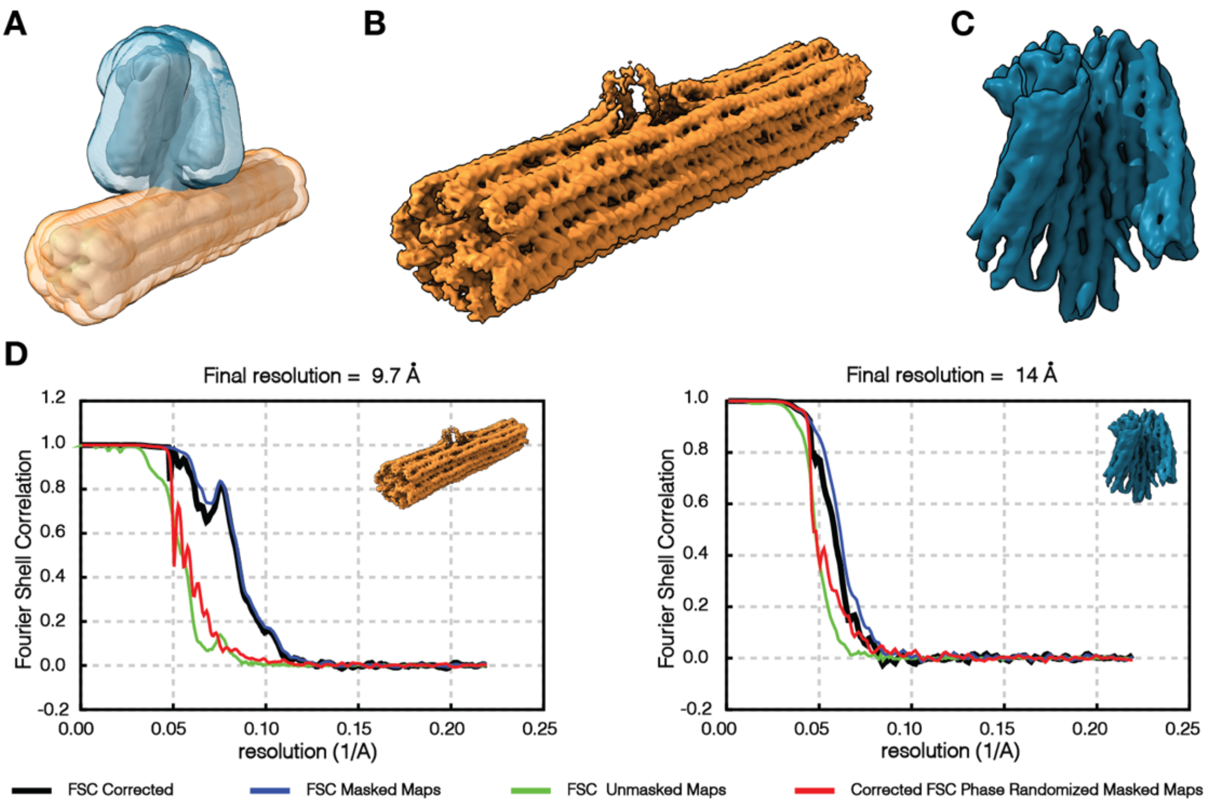

Figure S9. Multibody (MB) refinement-based reconstruction of the right-handed turbine variant.

(A) Masks of the rotor (blue transparent) and the lever (orange transparent) used for MB refinement. The map of the consensus refinement of the entire object is shown as a reference (gray). **(B)** Post-processed map of the MB-refined lever. **(C)** Post-processed map of the turbine reconstructed from a MB-refinement based set of partially-signal-subtracted particles. **(D)** FSC curves of the lever (left) and the turbine (right).

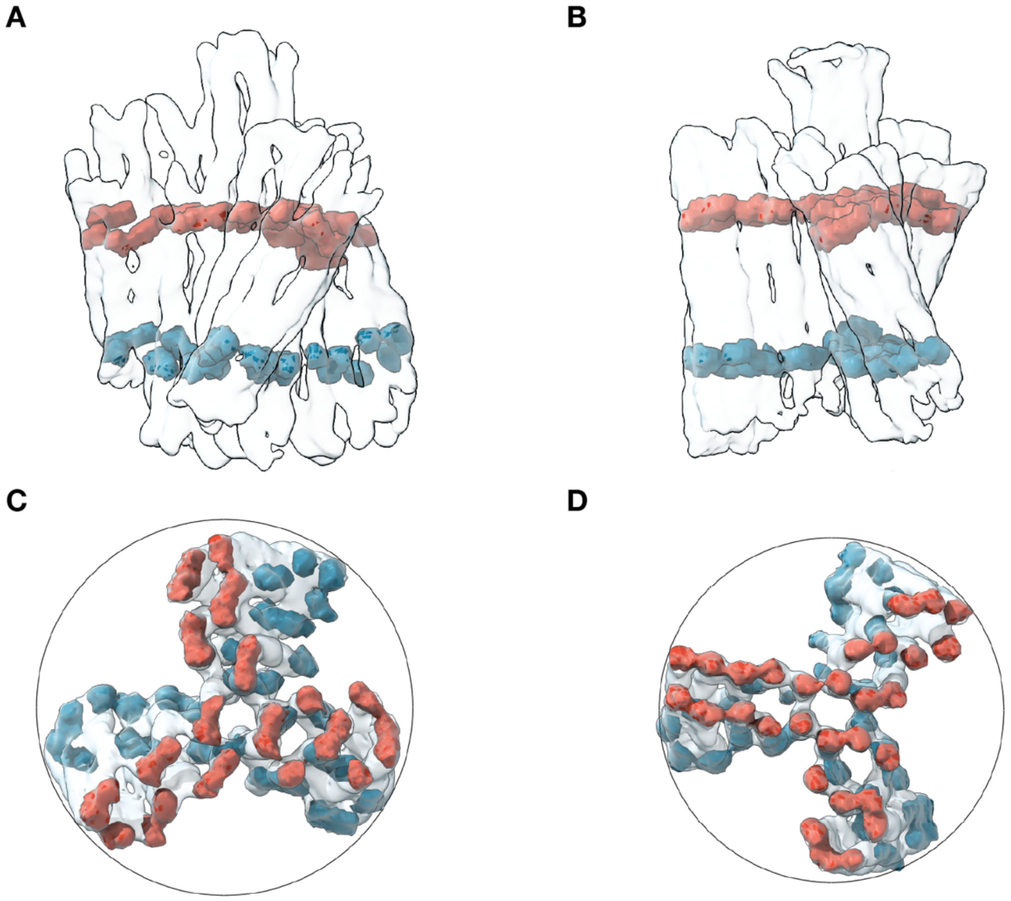

Figure S10. Twist measurement.
(A) Right-handed (RH) version of the turbine. The top (red) and bottom (blue) slice is extracted from the well-resolved central part of the map at base pair positions which are on the same plane in the design. **(B)** Like in A but for the left-handed (LH) version. **(C) – (D)** Top views of RH and LH versions. As the relative insertion density in the RH version is large than the relative deletion density in the LH version, the number of insertions is higher than the number of deletions (1 more vs 0.5 fewer bases compared to the regular 7 bp segments), and accordingly, a higher twist degree can be expected for the RH turbines.

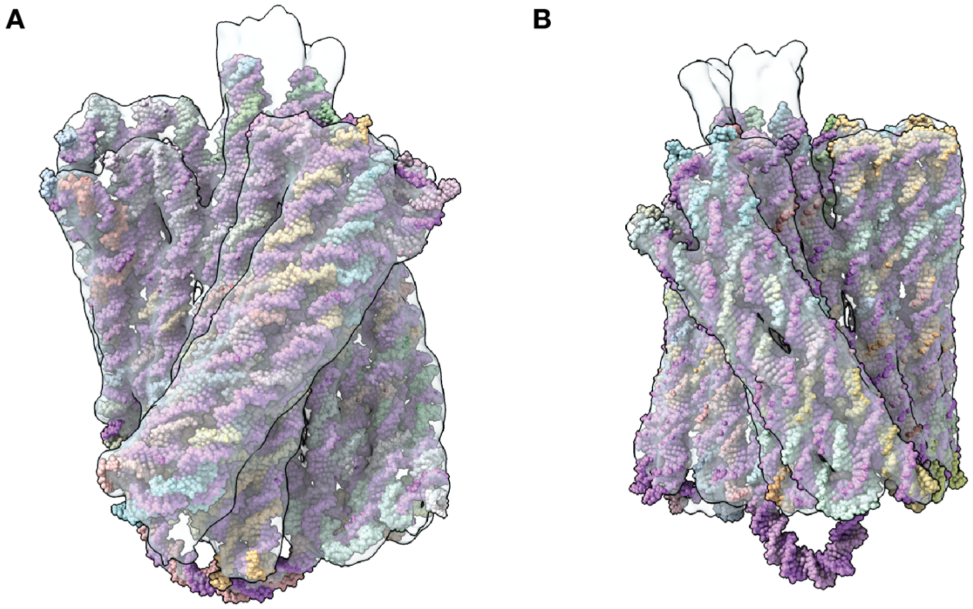

Figure S11. Atomic model fits.
(A) Right-handed version of the turbine. **(B)** Left-handed version of the turbine.

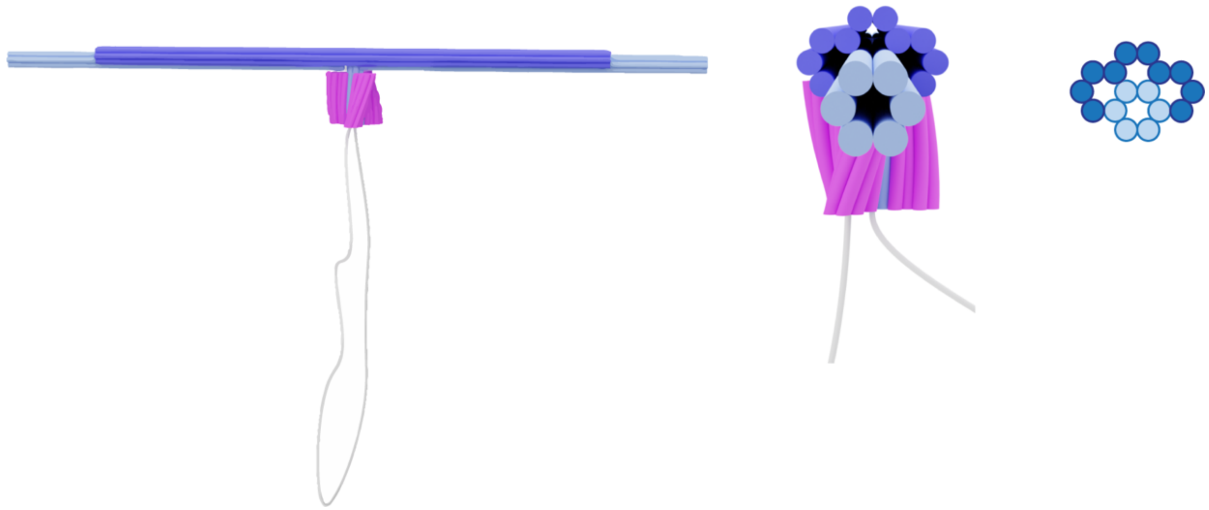

Figure S12. Schematics of the front (left) and sideview (right) of the turbine with the DNA bundle.

Note that middle 6hb part is reinforced with additional 10 helices (dark blue) to make it a 16hb structure.

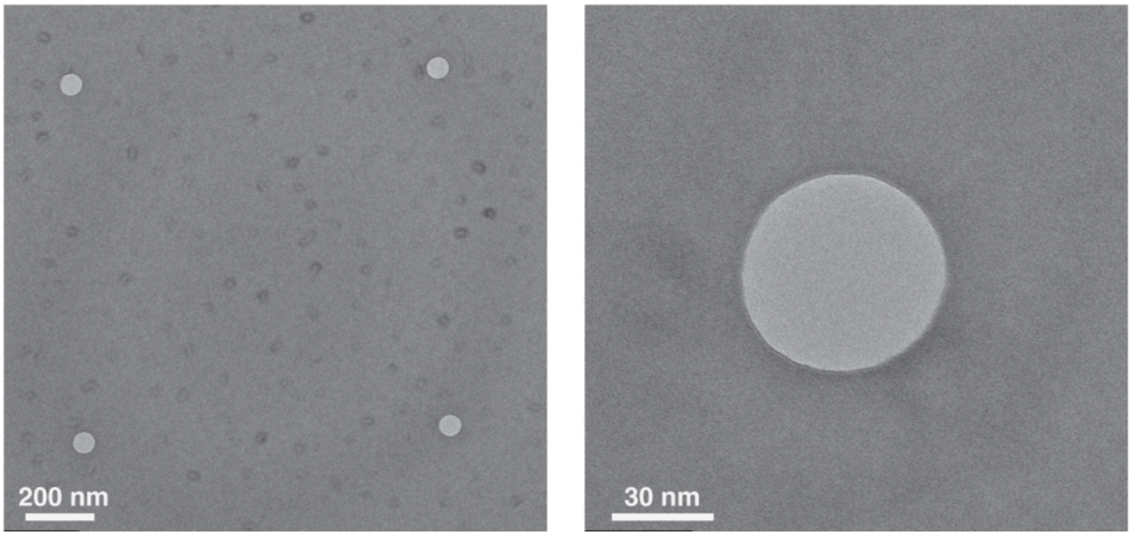

Figure S13. Typical TEM images of solid-state nanopores used in the experiments.

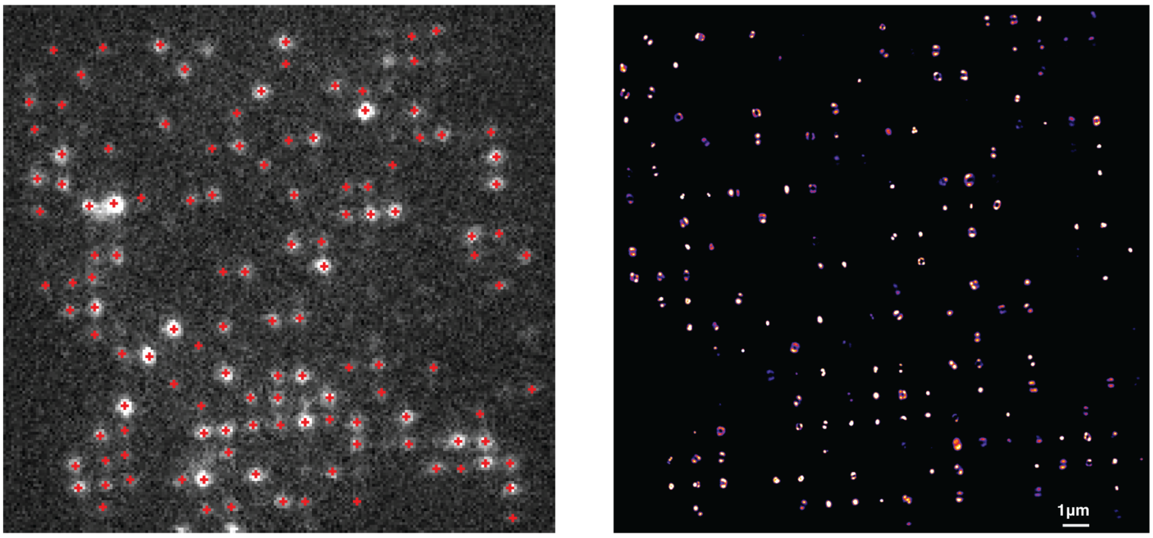

Figure S14. Example of the high-throughput fluorescence imaging of rotors in a nanopore array.

(Left) Example raw data frame of DNA turbines docked onto a nanopore array, and the corresponding single-particle localization results. Frame exposure time 5 ms. (Right) Results of the single-particle localization heatmap from 8000 stacked frames. Scalebar: 1 µm.

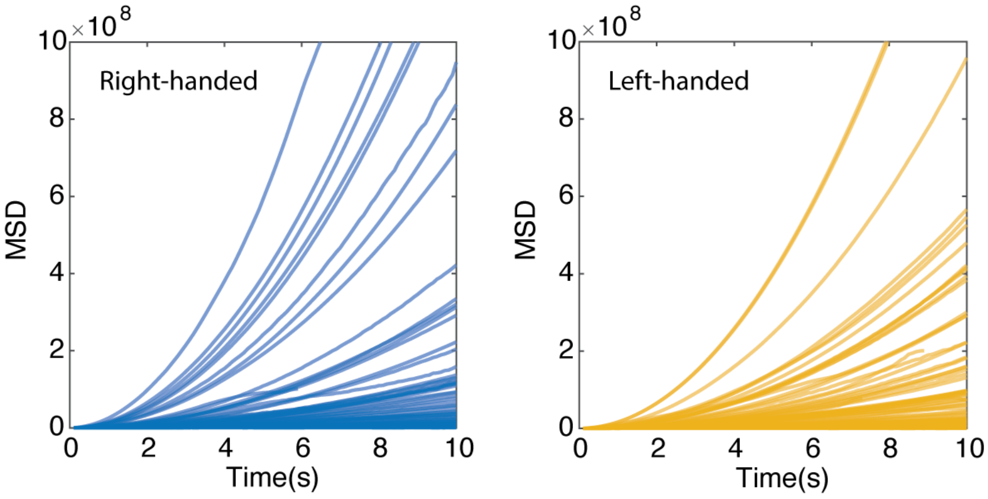

Figure S15. MSD plots corresponding to the trajectories shown in Fig. 2.

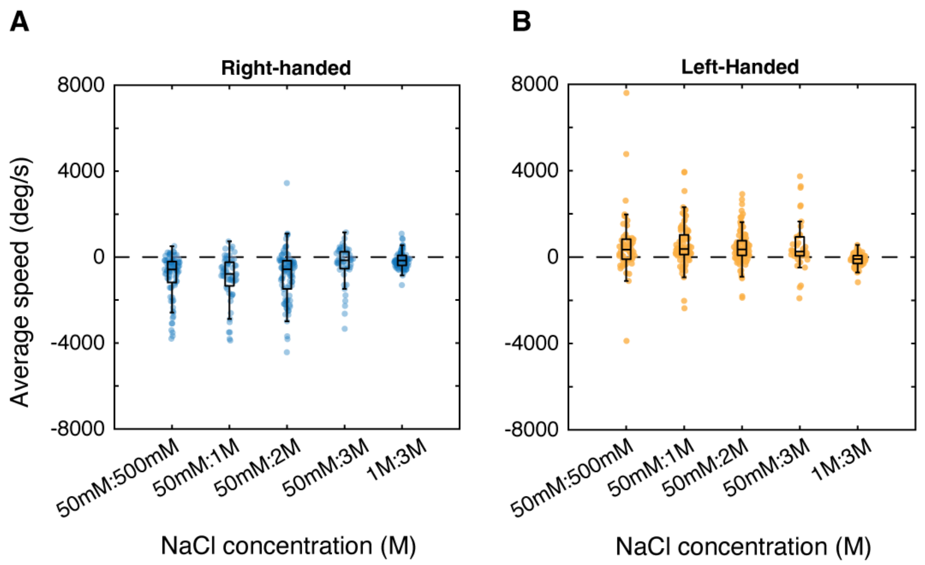

Figure S16. Average rotation speed of DNA bundle driven left-handed and right-handed turbine.

Data for different transmembrane NaCl concentration gradients, from left to right: 50mM:500 mM, 50mM:1M, 50mM:2M, 50mM:3 M, 1M:3 M (n_A= 124, 74, 116, 67, 154. n_B = 98, 141, 159, 80, 130). In all box plots: center line, median; box limits, upper and lower quartiles; whiskers, 1.5x interquartile range.

.

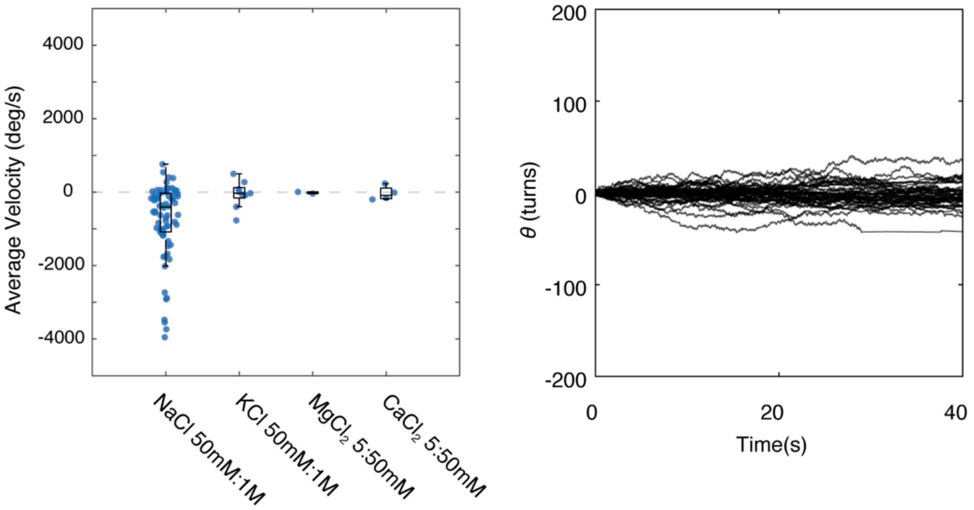

Figure S17. Rotations under different types of cation gradients.
Left: the calculated averaged velocity of turbines under 50mM:1M NaCl, 50mM:1M KCl, 5:50mM $MgCl_2$, and 5:50 mM $CaCl_2$, respectively (n = 74, 9, 2, and 4). Both divalent cation gradient conditions contain 50 mM KCl (equal concentration on both sides of the membrane). Right: example angular displacement of turbine motions under 5:50mM $MgCl_2$. In all box plots: center line, median; box limits, upper and lower quartiles; whiskers, 1.5x interquartile range.

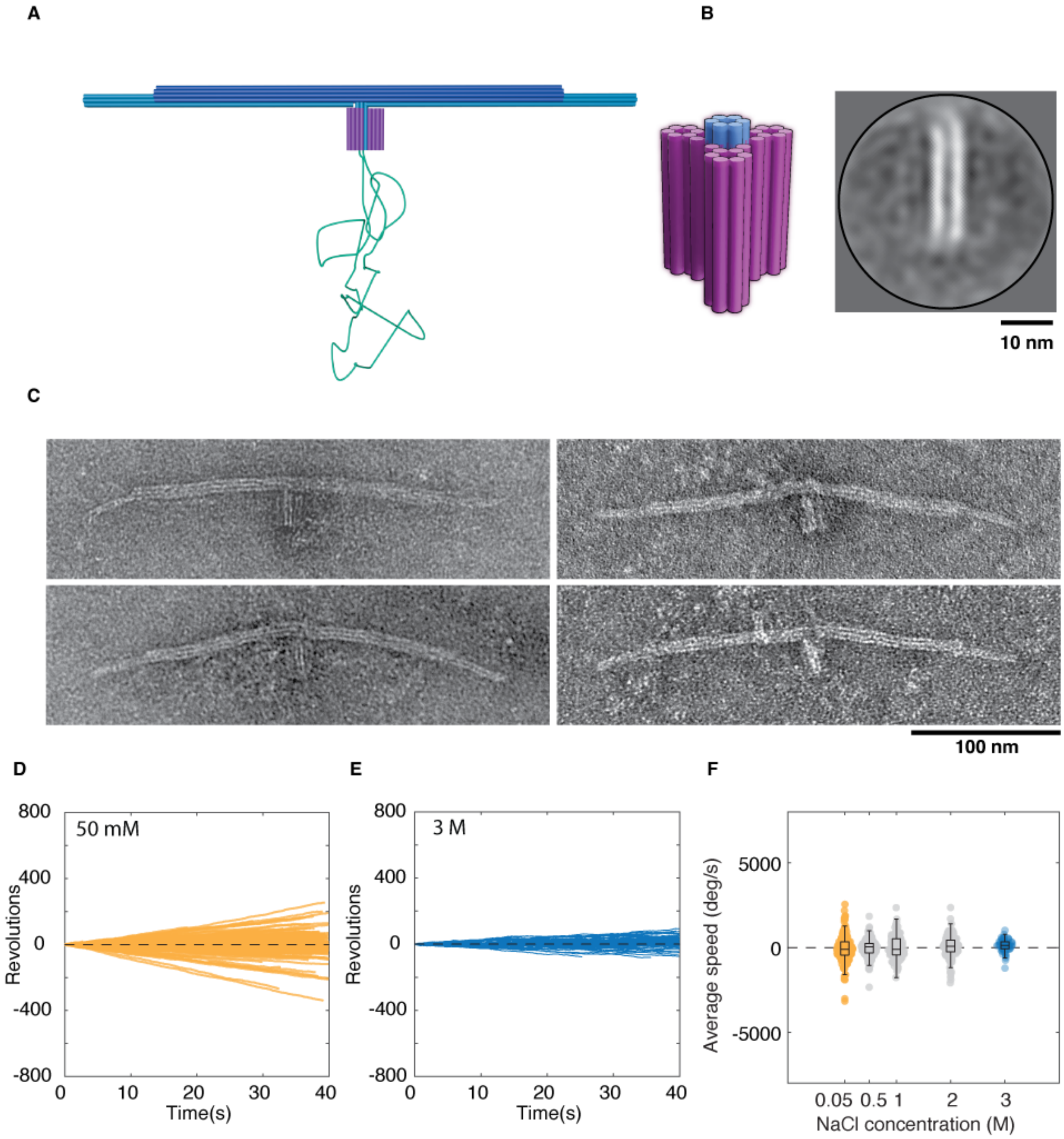

Figure S18. Achiral turbines did not show a directional preference.
(A) Schematic of the achiral turbine with load as a control structure. **(B)** Class-averaged negative stained TEM images of the achiral turbine structure with corresponding schematics. **(C)** Negative stained TEM images of the achiral turbine structure. **(D)** Example cumulative angular-displacement curves for achiral turbines under a 100-mV bias voltage in 50 mM NaCl (n = 259) and **(E)** in 3 M NaCl (n = 96). **(F)** Average rotation speed for achiral turbines in NaCl concentrations of 50 mM, 500 mM, 1M, 2M, 3M (n = 246, 250, 161, 173, and 90, respectively). In all box plots: center line, median; box limits, upper and lower quartiles; whiskers, 1.5x interquartile range.

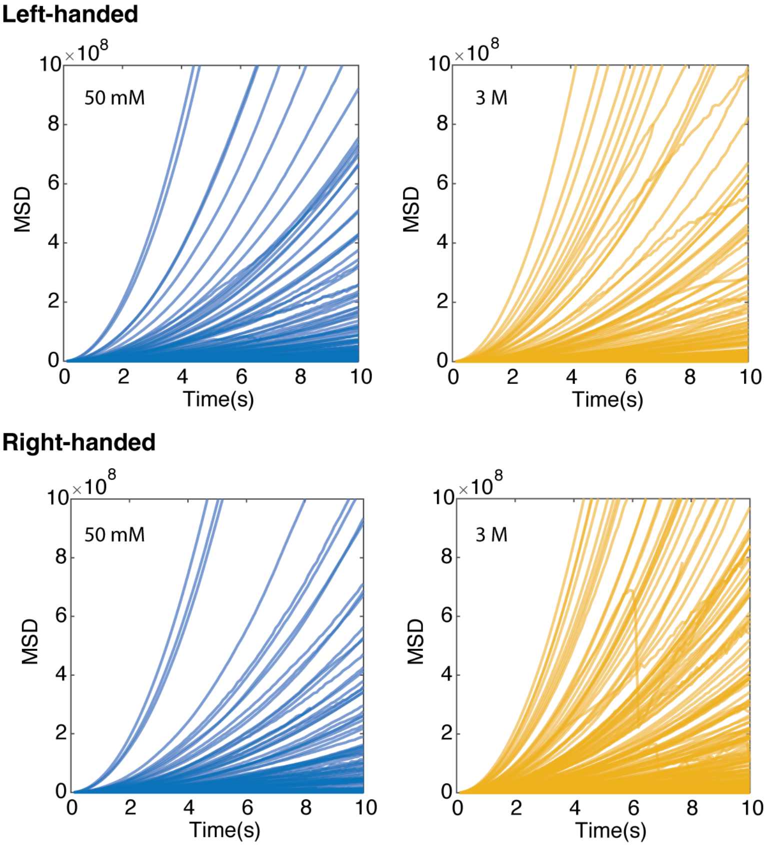

Figure S19. MSD plots corresponding to the trajectories shown in Fig. 3.

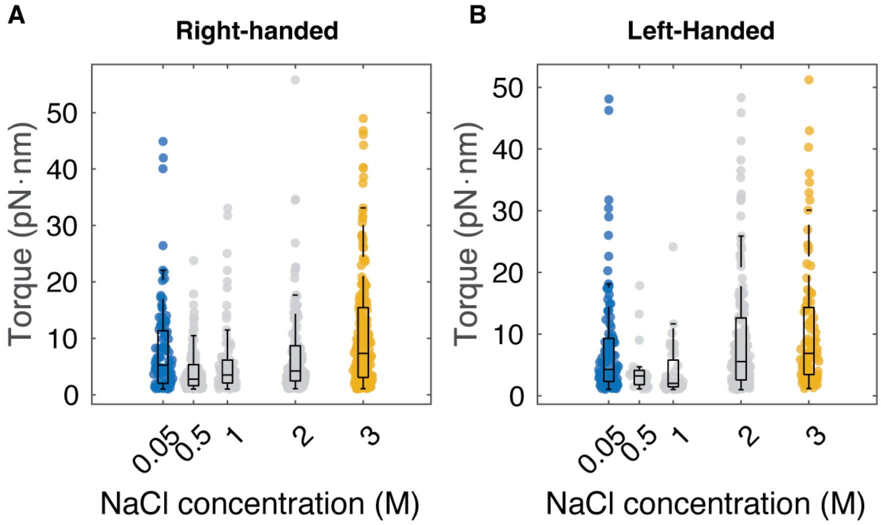

Figure S20. Estimated torques for right-handed (A) and left-handed (B) DNA turbines. All estimations result from the data shown in Fig. 3f and 3i (with the same sample sizes), obtained under 100 mV bias voltage. In all box plots: center line, median; box limits, upper and lower quartiles; whiskers, 1.5x interquartile range.

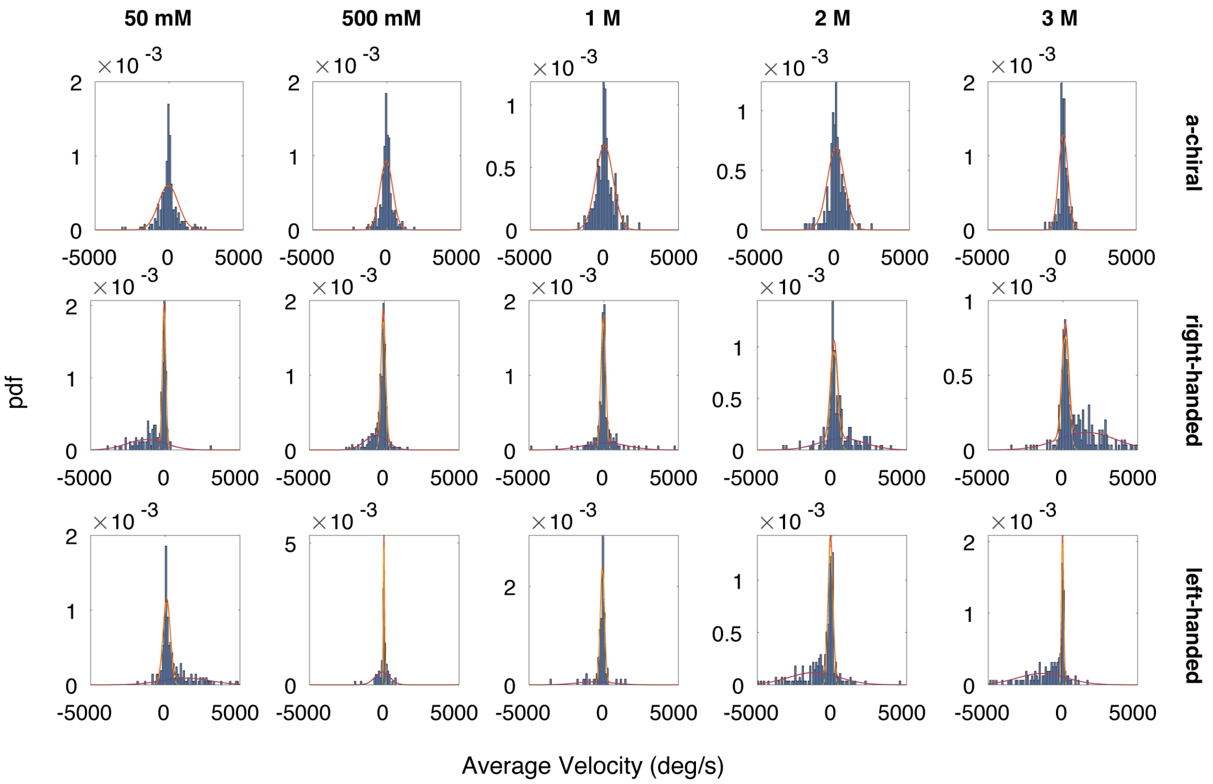

Figure S21. Average velocity distribution and model fitting for rotation data from achiral (1st row), right-handed (2nd row), and left-handed (3rd row) structures.

Each vertical column corresponds to one NaCl concentration (left to right): 50 mM, 500mM, 1M, 2M, 3M. For achiral structures (1st row), a single normal distribution was fitted to the data in all conditions. For chiral structures (2nd – 3rd rows), the sum of two normal distributions was fitted to the data, with one peak with a mean velocity of approximately zero (which are presumably due to nonfunctional structures because of nonspecific interaction with the surface, failure of the structural integrity, or incorrect docking), as well as one with a mean finite velocity.

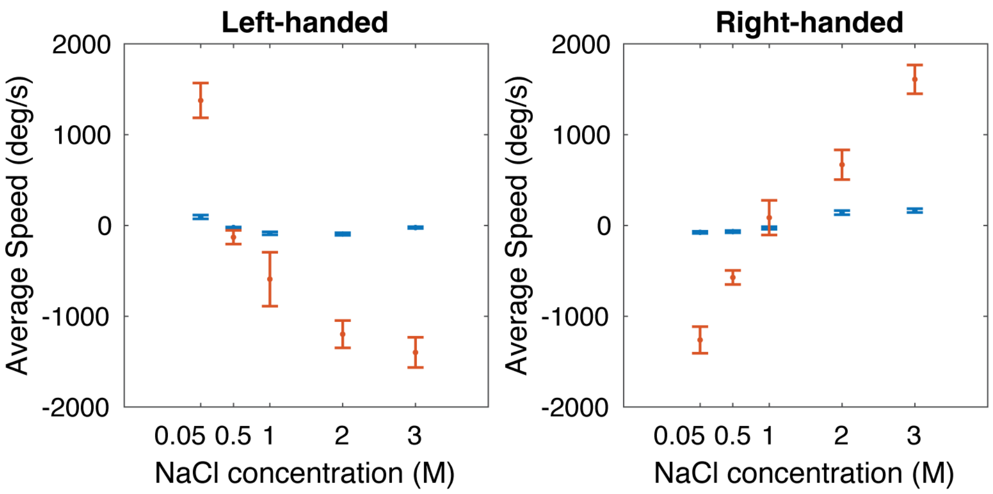

Figure S22. Mean speeds of left- and right-handed structures from the fitting results in Fig. S20.

The mean value of the peak near zero is marked in blue, and seen to be located near zero, as expected. The mean value of the second peak is marked in red (and shown in Fig. 3g and 3m in the main text, same sample sizes as Fig. 3f and 3l). Error bars indicate the standard error of the mean.

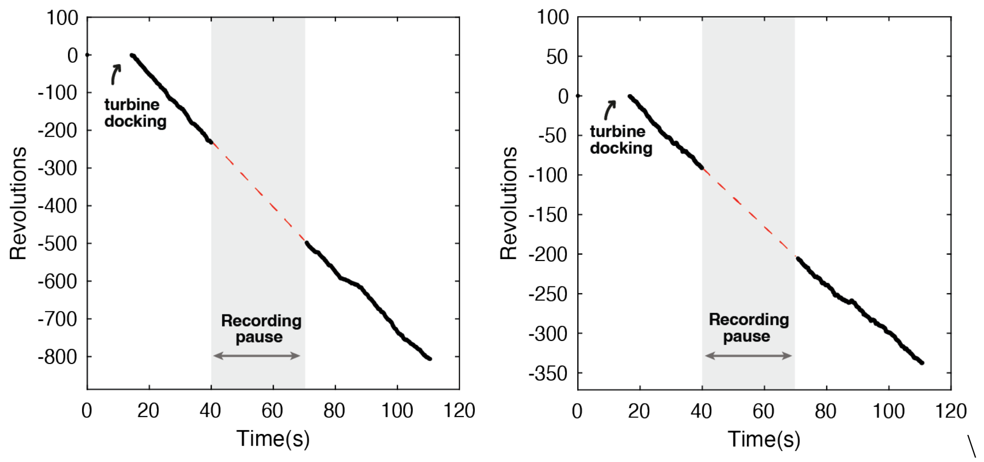

Figure S23. Examples of rotations after 40 seconds.

The recording was paused after the first 40-second period and restarted after 30 seconds. The starting Y value of each 2nd recording is the extrapolation of the first measurement segment (red dashed line). Data were recorded from the left-handed turbines, 3M NaCl, driven by 100 mV voltage.

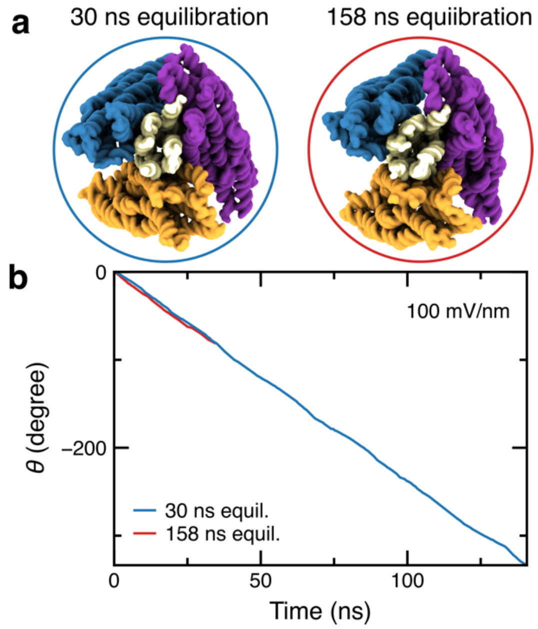

Figure S24. Control simulation of alternative initial turbine configuration. (a) For the results reported in the main text, the configuration of the turbine in 50 mM NaCl was frozen by an RMSD restraint (see Methods for details) after 30 ns of equilibration (left). A second system was constructed with RMSD restraint freezing the microscopic configuration of the rotor after 158 ns of equilibration (right). (**b**) Subject to an applied electric field, the two turbine configurations were found to rotate at a nearly identical rate.

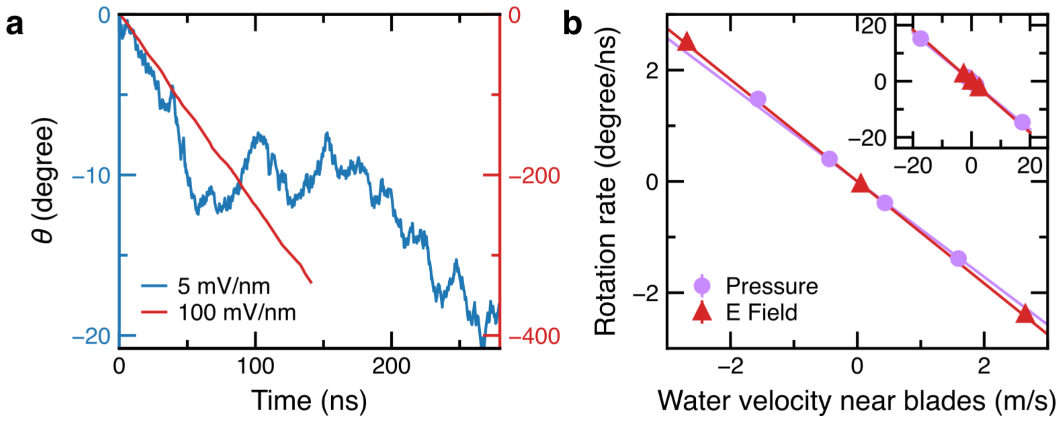

Figure S25. Simulated rotation of turbine in 50 mM NaCl under various conditions. (a) Rotation of the turbine at 5 mV/nm (blue; left axis) or 100 mV/nm (red; right axis) applied bias. (**b**) Rotation rate versus local water velocity near the turbine.

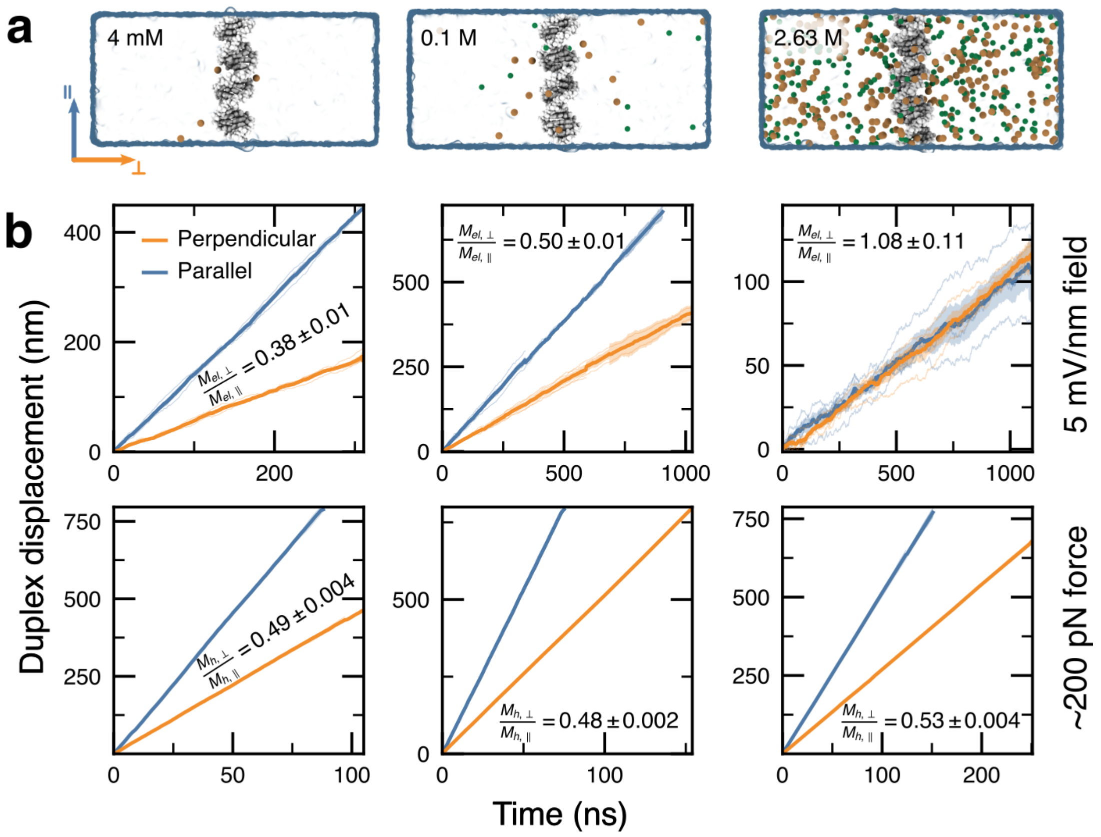

Figure S26. Dependence of DNA helix mobility on salt concentration in MD simulations. **(a)** Three systems containing a single DNA helix in electrolyte solution with 10% of ions depicted. (**b**) Center-of-mass displacement of the DNA helix relative to the water when driven by an electric field (top row) or force (bottom row) applied parallel (blue) or perpendicular (orange) to the DNA axis. For each condition, four simulations were performed (thin lines), allowing the average displacement (thick lines) and the standard error of the mean (shaded region) to be computed at each frame of the trajectory. The mobility was estimated for each replicate simulation from a linear regression, yielding the annotated perpendicular-to-parallel mobility ratio and the standard error. Top row: a 5 mV/nm field drove displacement. Bottom row: a force applied to the DNA (104, 105, and 114 pN in systems representing 0.004 , 0.1, and 2.6 M conditions, respectively) with an equal and opposite force distributed over the oxygen atoms of the water. A momentum-conserving Lowe-Andersen thermostat held the temperature near 295 K during the simulations.

Supplementary Movie S1: MD simulation of the right-handed DNA turbine rotation in 100 mM NaCl condition under electric field, as shown in Fig 4.

Supplementary Movie S2: MD simulation of the right-handed DNA turbine rotation in 3 M NaCl condition under electric field, as shown in Fig 4.

Supplementary References

1 Tirado, M. M. & De La Torre, J. G. Rotational dynamics of rigid, symmetric top macromolecules. Application to circular cylinders. *The Journal of Chemical Physics* **73**, 1986-1993 (1980).

2 Yariv, E. & Schnitzer, O. The electrophoretic mobility of rod-like particles. *Journal of Fluid Mechanics* **719**, R3 (2013).
